# Structural study reveals the temperature-dependent conformational flexibility of Tk-PTP, a protein tyrosine phosphatase from *Thermococcus kodakaraensis* KOD1

**DOI:** 10.1371/journal.pone.0197635

**Published:** 2018-05-23

**Authors:** Hye-Yeoung Yun, Jinhyuk Lee, Hyunmin Kim, Hyojung Ryu, Ho-Chul Shin, Byung-Ha Oh, Bonsu Ku, Seung Jun Kim

**Affiliations:** 1 Disease Target Structure Research Center, Korea Research Institute of Bioscience and Biotechnology, Daejeon, Republic of Korea; 2 Department of Bioscience, University of Science and Technology KRIBB School, Daejeon, Republic of Korea; 3 Korean Bioinformation Center, Korea Research Institute of Bioscience and Biotechnology, Daejeon, Republic of Korea; 4 Department of Biotechnology, University of Sciences and Technology KRIBB School, Daejeon, Republic of Korea; 5 Department of Biological Sciences, KAIST Institute for the Biocentury, Korea Advanced Institute of Science and Technology, Daejeon, Republic of Korea; Russian Academy of Medical Sciences, RUSSIAN FEDERATION

## Abstract

Protein tyrosine phosphatases (PTPs) originating from eukaryotes or bacteria have been under intensive structural and biochemical investigation, whereas archaeal PTP proteins have not been investigated extensively; therefore, they are poorly understood. Here, we present the crystal structures of Tk-PTP derived from the hyperthermophilic archaeon *Thermococcus kodakaraensis* KOD1, in both the active and inactive forms. Tk-PTP adopts a common dual-specificity phosphatase (DUSP) fold, but it undergoes an atypical temperature-dependent conformational change in its P-loop and α4−α5 loop regions, switching between the inactive and active forms. Through comprehensive analyses of Tk-PTP, including additional structural determination of the G95A mutant form, enzymatic activity assays, and structural comparison with the other archaeal PTP, it was revealed that the presence of the GG motif in the P-loop is necessary but not sufficient for the structural flexibility of Tk-PTP. It was also proven that Tk-PTP contains dual general acid/base residues unlike most of the other DUSP proteins, and that both the residues are critical in its phosphatase activity. This work provides the basis for expanding our understanding of the previously uncharacterized PTP proteins from archaea, the third domain of living organisms.

## Introduction

Protein tyrosine phosphatases (PTPs) constitute a protein family, which mediates one of the common and significant post-translational modifications called protein tyrosine phosphorylation by coordinating with protein tyrosine kinases [1–4]. PTP proteins can be classified into several categories based on folding and domain composition. Dual-specificity phosphatases (DUSPs) comprise one such subfamily of PTP proteins, which are characterized by distinguishing protein folding and dephosphorylating ability. DUSPs are composed of a core region containing a five-stranded β-sheet rounded by four α–helices and mid-domain variations [5]. They have an ability to remove a phosphate group from not only phosphorylated tyrosine but also phosphorylated serine/threonine [6]. DUSP proteins play a crucial role in the regulation of diverse intracellular or intercellular signaling and other biological processes, including cell differentiation and proliferation, programmed cell death, and vesicular trafficking [6,7]. Structural studies revealed that PTP proteins adopting the DUSP fold, which was defined by the comprehensive structural analysis of the human DUSP proteins as to be composed of highly conserved “core region” containing four α-helices and five β-strands and diverse “mid-domain variations” [5], are found across a variety of species, including eukaryotes such as humans [5], bacteria [8,9], viruses [10,11], and the archaeon, *Sulfolobus solfataricus* [12]. Even though a great number of the PTP family proteins have been under intensive structural investigation, the *S*. *solfataricus* PTP protein, usually called as SsoPTP, is the sole archaeal PTP protein whose structure has been previously determined, restricting our understanding of PTPs from the archaeal domain.

*Thermococcus kodakaraensis* KOD1 is a hyperthermophilic archaeon, which grows in the temperature range of 60–100°C. It was first isolated from a solfatara near the shore of Kodakara Island, Japan [13,14]. The genome of *T*. *kodakaraensis* KOD1 contains 2088 kilo base pairs that encode more than 2300 proteins [14]. It was reported by Jeon *et al*. that this archaeon has a PTP protein, Tk-PTP, which contains a PTP signature motif (HCxxGxxR; HC^93^MGGLGR^99^ in Tk-PTP) and exhibits phosphatase activity towards phosphotyrosine and phosphoserine that was optimal at 80°C [15]. It was also suggested that this protein might have a residue that functionally substitutes the highly conserved general acid/base residue (also conserved in Tk-PTP as Asp63), which is critical for the enzymatic activity of most PTP proteins, as the alanine mutation of this residue in Tk-PTP did not appear to impair its dephosphorylating activity [15]. This hypothesis was intriguing, but could not be corroborated due to the lack of structural analysis of this protein. In this study, we report the crystal structures of three forms of Tk-PTP, which showed that this protein forms a canonical DUSP fold and that it undergoes an atypical temperature-dependent conformational change between two forms. Structural and biochemical analyses revealed that this structural alteration requires the presence of the GG motif in the P-loop, and that Tk-PTP has dual amino acids, Asp63 and Glu132, both functioning as the general acid/base residues. We also provide the sequence and structural comparisons between the two archaeal PTP proteins, SsoPTP and Tk-PTP. These results expand our understanding of structural and biochemical features of PTP proteins from archaea that are poorly understood.

## Results

### Structural determination of Tk-PTP(form I)

**Fig 1:**
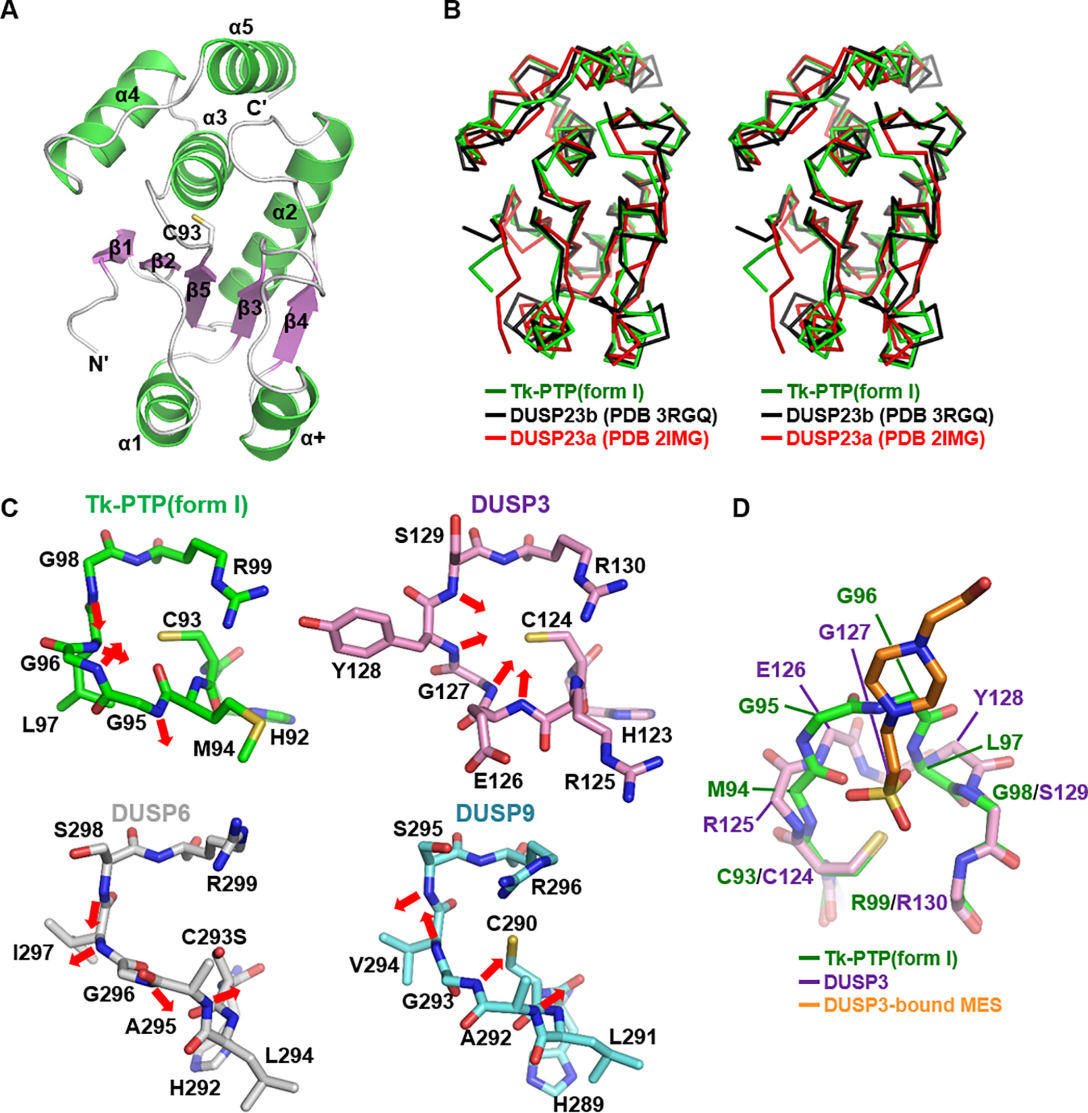
Crystal structure of Tk-PTP(form I). (A) Tk-PTP(form I) is presented as ribbon drawings with secondary structure labels. Green, α-helices; violet, β-strands; white, the remaining structures. The catalytic cysteine residue shown as a stick model is labeled as C93. (B) Stereo views of C_α_ traces of three superimposed DUSP proteins. (C) The P-loops of four DUSP proteins are shown in labeled sticks and compared. Arrows indicate the direction of main chain amides of four P-loop-constituting central residues. The PDB codes are 1VHR for DUSP3, 1MKP for DUSP6, and 3LJ8 for DUSP9, respectively. (D) Structural alignment of the P-loops of Tk-PTP(form I) and MES-bound DUSP3 shown in sticks.

The recombinant Tk-PTP protein expressed at 25°C in *Escherichia coli* was purified at room temperature using a Ni–NTA column and size-exclusion chromatography, which was subsequently subjected to crystallization trials at 18°C. Triangular prism-shaped crystals were obtained, which were used for determination of the crystal structure of Tk-PTP to a resolution of 1.7 Å, referred to as Tk-PTP(form I) in this manuscript (Table 1). The asymmetric unit of the crystal (space group *P*2_1_2_1_2_1_) contains one molecule of Tk-PTP(form I), in which a central β-sheet constituted by five β-strands [β1(Lys6−Asp9), β2(Val12−Ser15), β3(Ala32−Val34), β4(Glu55−His58), and β5(Lys88−His92)] is sandwiched by four helices [α2(Val69−Glu85), α3(Gly98−Arg112), α4(Leu116−Lys126), and α5(Gln134−Arg146)] on one side, and by two helices [α1(Glu22−Asp29) and α+(Leu46−Arg52)] on the other (Fig 1A). The overall structure indicated that Tk-PTP(form I) adopts the typical DUSP fold, composed of a core region (β1–β5 and α2–α5) and mid-domain variations (α1 and α+) [5]. A search for homologous protein structures using the Dali server [16] identified a number of mammalian DUSP proteins showing high structural similarity with Tk-PTP(form I), including DUSP23b (PDB code 3RGQ; Z-score 22.1), DUSP23a (PDB code 2IMG; Z-score 20.7), DUSP5 (PDB code 2G6Z; Z-score 19.7), and DUSP7 (PDB code 4Y2E; Z-score 19.5). Upon superimposition, we found that Tk-PTP(form I) was well aligned to DUSP23b and DUSP23a, with a root mean square deviation (RMSD) of 1.52 Å over 142 aligned residues and 1.54 Å over 137 aligned residues, respectively (Fig 1B). This also supports the notion that Tk-PTP(form I) adopts a canonical DUSP fold.

**Table 1:** Data collection and structure refinement statistics.

Data Collection	Tk-PTP(form I)	Tk-PTP(form II)	Tk-PTP(G95A)
**Space group**	*P*2_1_2_1_2_1_	*P*6_1_	*H*3
**Unit cell dimensions**			
**a, b, c (Å)**	29.5, 50.6, 78.5	107.7, 107.7, 83.8	154.8, 154.8, 75.2
α**, β, γ (**^**o**^**)**	90, 90, 90	90, 90, 120	90, 90, 120
**Resolution (Å)**	50.0−1.7 (1.73−1.70)^b^	50.0−1.8 (1.83−1.80)^b^	50.0−2.3 (2.34−2.30)^b^
***R***_**sym**_^**a**^ **(%)**	5.3 (19.8)	5.6 (27.3)	4.9 (19.9)
***I*/σ(*I*)**	46.0 (7.2)	31.6 (4.8)	27.0 (4.1)
**Completeness (%)**	97.2 (92.7)	97.6 (94.9)	95.2 (91.7)
**Redundancy**	5.4	4.7	4.3
**Refinement**			
**Resolution (Å)**	50.0−1.7	50.0−1.8	50.0−2.3
**Number of reflections**	13084	45502	27128
***R***_**work**_^**c**^**/*R***_**free**_ **(%)**	18.6 / 22.7	17.4 / 20.9	21.4 / 25.9
**Number of atoms**			
**Protein**	1183	3574	3707
**Ion**		12	11
**Water**	106	393	121
**R.m.s deviations**			
**Bond lengths (Å)**	0.007	0.008	0.009
**Bond angles (**^**o**^**)**	0.905	0.964	1.000
**Ramachandran plot (%)**			
**Most favored region**	96.5	94.7	94.7
**Additionally allowed region**	3.5	4.6	4.6
**Outliers**	0.0	0.7	0.7
**Average B-values (Å**^**2**^**)**			
**Protein**	23.4	25.1	38.3
**Ion**		20.1	48.9
**Water**	30.5	30.8	36.5

^a^*R*_sym_ = Σ |*I*_obs_—*I*_avg_| / *I*_obs_, where *I*_obs_ is the observed intensity of individual reflection and *I*_avg_ is the average over symmetry equivalents.

^b^The numbers in parentheses are statistics from the shell with the highest resolution.

^c^*R*_work_ = Σ ||*F*_o_|—|*F*_c_|| / Σ |*F*_o_|, where |*F*_o_| and |*F*_c_| are the observed and calculated structure factor amplitudes, respectively. *R*_free_ was calculated with 10%, 4.39%, and 7.37% of the Tk-PTP(form I), Tk-PTP(form II), and Tk-PTP(G95A) data, respectively.

### Structural analysis of the conformation of the P-loop in of Tk-PTP(form I)

PTP proteins commonly share a highly conserved active site motif called the PTP signature motif, which is also present in Tk-PTP as HC^93^MGGLGR^99^. This motif, constituting the phosphate binding loop (or simply called P-loop), contains the catalytic cysteine residue (Cys93) that functions as a nucleophile for dephosphorylation and the conserved arginine residue (Arg99) that anchors the phosphate group of the substrate during the enzyme reaction. The residue next to the indicated arginine is also conserved in a number of, but not all, PTP proteins as serine or threonine, which stabilizes the catalytic cysteine thiolate via a hydrogen bond: Ser100 in Tk-PTP appears to play the role, as its side chain hydroxyl group is in a contact with the side chain thiol of Cys93 in our structure (not shown). The proper arrangement of the backbone amides of the P-loop residues is also crucial for the enzyme activity of PTP proteins. These amide nitrogens, which have a partial positive charge because of the peptide bond resonance [17], are oriented towards the interior of the active site pocket; therefore, they create a positive electrostatic potential, which is not only complementary to the negatively charged substrate phosphate group but also lowers the p*K*a value of the catalytic cysteine residue [18,19]. Therefore, we analyzed the conformation of the P-loop of Tk-PTP(form I) by structurally comparing it to that of the other DUSP proteins. DUSP3 (also known as VHR), a DUSP protein with potent dephosphorylating activity [20,21], exhibits the canonical active P-loop conformation, in terms of the direction of the side chains of Cys124 and Arg130 as well as the main chain amides of Glu126−Ser129 (Fig 1C, right top). However, the P-loops of DUSP6 and DUSP9, which were reported to be weakly active [22–24], show impaired conformations (Fig 1C, bottom) that are quite different from that of DUSP3. Similarly, Tk-PTP(form I) adopts a distorted P-loop conformation in which the backbone amides of Gly95−Gly98 are oriented away from the active site pocket (Fig 1C, left top). Structural alignment between the P-loops of Tk-PTP(form I) and DUSP3 bound to 2-(N-morpholino)-ethanesulfonic acid (commonly called MES) shows that the main chain backbone of the residues Met94−Leu97 of Tk-PTP(form I) are arranged differently from that of the corresponding residues of DUSP3 (Fig 1D). Furthermore, steric hindrance occurs between Tk-PTP(form I) and the DUSP3-bound phosphotyrosine-mimicking molecule in the superposed model (Fig 1D), demonstrating that the active site motif of Tk-PTP(form I) is not suitable to accommodate a phosphotyrosine moiety; therefore, Tk-PTP(form I) should have limited phosphatase activity.

### Structural determination of Tk-PTP(form II)

DUSP6 and DUSP9 are members of the mitogen-activated protein kinase (MAPK) phosphatase (MKP) subfamily, and are known as MKP-3 and MKP-4, respectively. Enzymatic activity of the MKP proteins was reported to be robustly enhanced by the MAPK binding domain (MKB)-mediated interaction with MAPKs, such as p38α, c-Jun N-terminal kinase 1, and extracellular signal-regulated kinase 2, which is presumed to be accompanied by a conformational change in the P-loop region [22–26]. However, Tk-PTP does not contain a MAPK binding domain, indicating that a novel mechanism is necessary for Tk-PTP to be activated. A previous study on Tk-PTP reported that its enzymatic activity is enhanced by heat treatment, and the optimum temperature for its phosphatase activity is 80°C [15]. Therefore, we assumed that Tk-PTP might undergo a temperature-dependent structural alteration, which should occur especially in its P-loop region. To validate this hypothesis, 0.6 mM purified Tk-PTP protein in the final purification buffer containing 50 mM Tris-HCl (pH 7.5), 200 mM NaCl, and 2 mM dithiothreitol was heated at 60°C for 3 hours to induce its conversion to an enzymatically active form. Simultaneously, sodium vanadate, a widely used PTP inhibitor targeting the catalytic site pocket, was added at a molar ratio of 1:3, to prevent this protein from reverting to the catalytically inactive form during crystallization. Hexagonal prism-shaped crystals were obtained with the heat-treated Tk-PTP protein, and its structure was determined to a resolution of 1.8 Å (Table 1), referred to as Tk-PTP(form II) in this manuscript. Three molecules of Tk-PTP(form II), which were well matched with each other when superimposed with an RMSD of 0.36−0.38 Å, were contained in the asymmetric unit of the crystal (space group *P*6_1_).

### Conformational difference between the P-loop of the two forms of Tk-PTP

**Fig 2:**
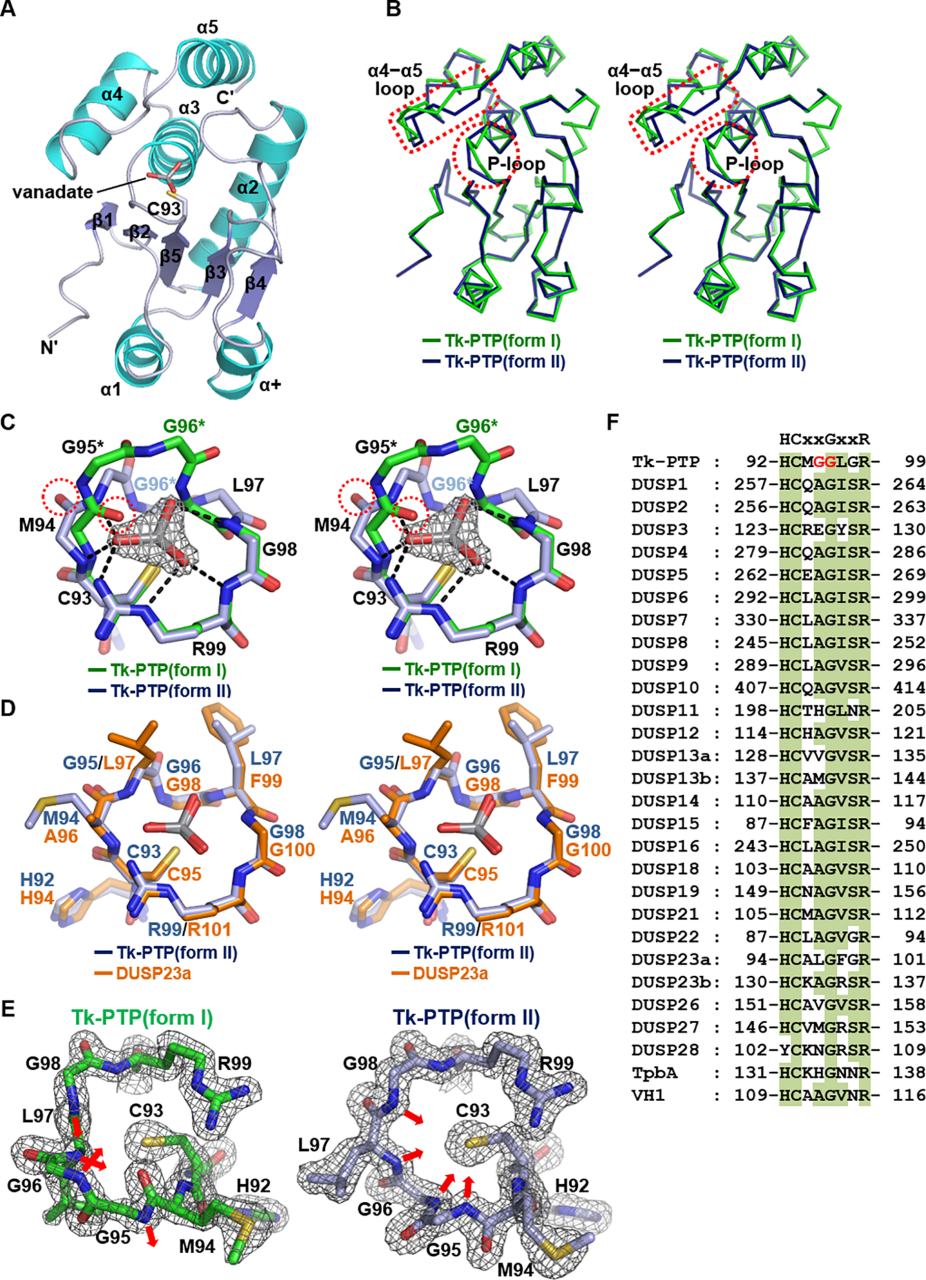
Crystal structure of Tk-PTP(form II). (A) Tk-PTP(form II) is presented as a secondary structure-labeled ribbon drawing. Cyan, α-helices; navy, β-strands; white, the remaining structures. The catalytic cysteine residue is labeled as C93 and the vanadate molecule bound to the active site pocket is shown in sticks. (B) Stereo views of C_α_ traces of two forms of Tk-PTP. The P-loop and α4−α5 loop regions are indicated by dotted circles and rectangles, respectively. (C) Stereo views of the superimposed P-loops of two forms of Tk-PTP shown in sticks. For clarity, the main chain and the side chains of Cys93 and Arg99 are shown. Vanadate-mediated hydrogen bonds and electrostatic interactions are presented as dashed lines. Dotted circles indicate the backbone carbonyl of Met94 that undergoes a peptide flip. The GG motif residues are marked with asterisks. A 2Fo-Fc electron density omit map of the vanadate molecule contoured at 1.5 σ is shown together. The temperature factor of vanadate bound to Tk-PTP(form II) is 18.2. (D) Stereo views of the superimposed P-loops of Tk-PTP(form II) and DUSP23a shown in sticks. The PDB code for DUSP23a bound to vanadate is 4ERC. (E) The P-loops of two forms of Tk-PTP are shown in labeled sticks along with the 2Fo-Fc electron density map contoured at 1.5 σ. The direction of main chain amides of four P-loop-constituting central residues are indicated by arrows. (F) The sequences of the P-loop of Tk-PTP and 28 DUSP members are aligned. DUSP1–28 are from human; TbpA is from *Pseudomonas aeruginosa*; VH1 is from *Vaccinia virus*. Conserved residues are shaded in green. The PTP signature motif is shown at the top, and the GG motif residues in the P-loop of Tk-PTP are marked in red.

The overall structure of Tk-PTP(form II), also adopting the canonical DUSP fold (Fig 2A), is nearly the same as that of Tk-PTP(form I). The two Tk-PTP structures overlapped well with each other with an RMSD of 0.70 Å over 144 structurally aligned residues (Fig 2B). However, the P-loop conformation of Tk-PTP(form II) is quite different from that of Tk-PTP(form I) (an RMSD of 2.39 Å over 8 aligned C_α_ atoms), with a flip of the Met94−Gly95 peptide bond and a remarkable shift in the main chain backbone atoms of Met94−Leu97 (Fig 2C). Vanadate is anchored in the active site of Tk-PTP(form II), stabilized by electrostatic interaction with the guanidinium group of Arg109 and by its oxygen atom-mediated hydrogen bonds with the main chain amides of Met94, Gly95, Leu97, Gly98, and Arg99 (Fig 2C). The Tk-PTP(form II) P-loop is structurally similar to those of catalytically active DUSP proteins such as DUSP23a (an RMSD of 0.29 Å over 8 aligned C_α_ atoms; Fig 2D). Consistently, the main chain amides of the P-loop of Tk-PTP(form II) are directed towards the interior of the active site pocket, similar to those of DUSP3 (Fig 1C), but remarkably differently to those of Tk-PTP(form I) (Fig 2E). These results collectively indicate that Tk-PTP has a unique P-loop, which is flexible and thus able to adopt both catalytically active and inactive conformations depending on the conditions. Such flexibility seems to have resulted from the presence of nonconserved glycine residue (Gly95 of Tk-PTP) at the fourth position in the PTP signature motif, which is usually (19 of 28) occupied by alanine in DUSP proteins (Fig 2F). Together with the highly conserved glycine at the fifth position (Gly96 of Tk-PTP), a unique glycine−glycine motif (referred to as the GG motif) is present in Tk-PTP (Fig 2F, marked in red). This motif showed a remarkable conformational difference between the two Tk-PTP structures when they were superimposed, with a distance of 2.5 Å between the two Gly95 C_α_ atoms and 5.9 Å between the two Gly96 C_α_ atoms (Fig 2C). Accordingly, the ϕ and ψ dihedral angles of those glycine residues were quite different in the two structures (S1 Fig). Notably, the dihedral angle of Gly95 of Tk-PTP(form I) is in the region that is unfavorable for most amino acids except for glycine on the Ramachandran plot (S1 Fig), implying that the indicated conformational conversion of the catalytic loop is only possible in a DUSP protein containing the GG motif in its P-loop.

### Structural alteration between the α4−α5 loop regions of the two forms of Tk-PTP

**Fig 3:**
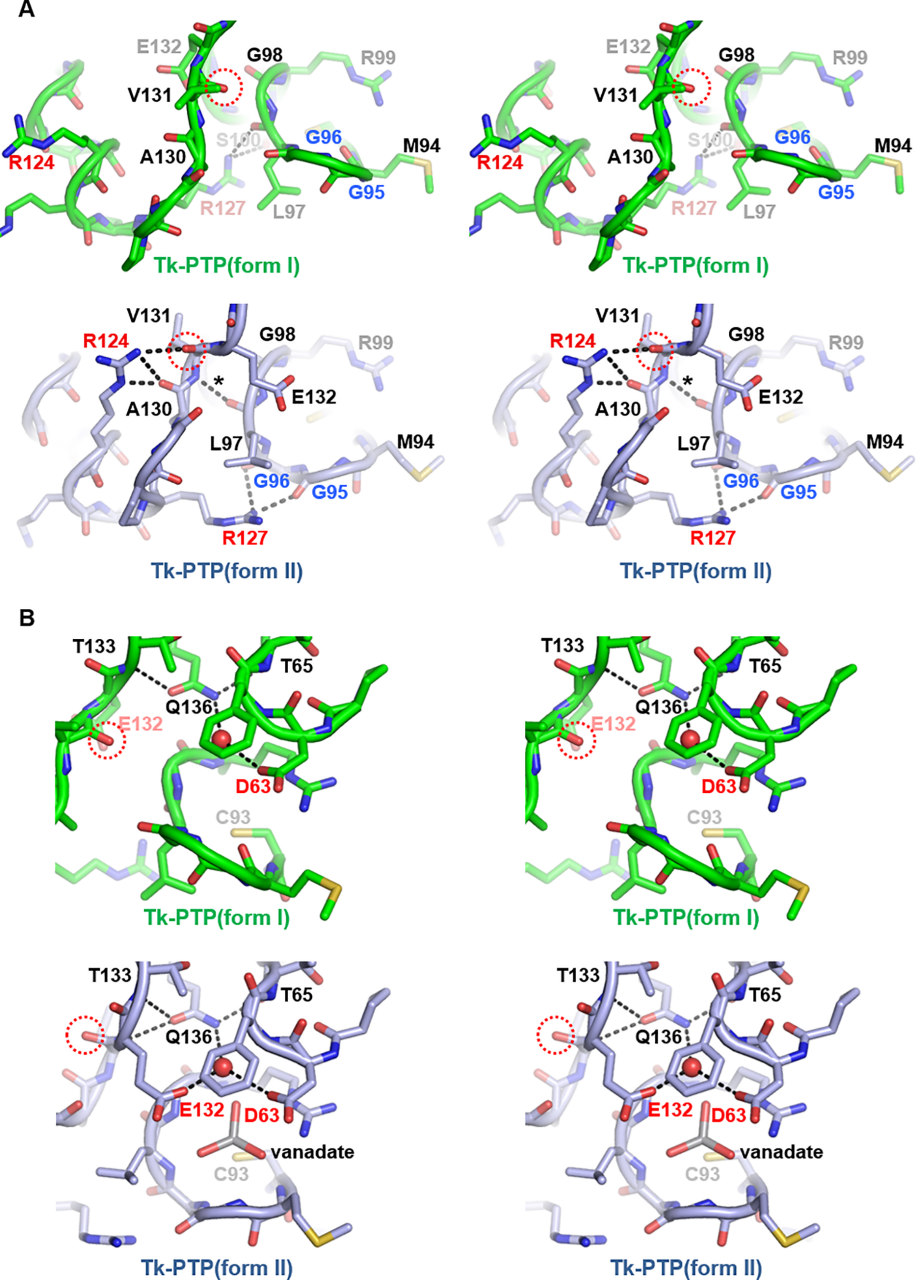
Structural comparison between two forms of Tk-PTP. Stereo views of two forms of Tk-PTP for structural comparison. Dotted circles indicate the backbone carbonyl of Val131 undergoing a peptide flip. Hydrogen bonds described in the main text are presented as dashed lines. (A) The P-loop and α4−α5 loop of two forms of Tk-PTP are structurally compared. The GG motif residues are labeled in blue, while Arg124 and Arg127 are labeled in red. (B) The conformation of dual general acid/base residues (labeled in red) are structurally compared between two forms of Tk-PTP.

Along with the conformational change of the P-loop region, an additional noticeable structural alteration between the two forms of Tk-PTP was observed in the α4−α5 loop region, which is in contact with the catalytic P-loop region (Fig 3A). This conformational change includes a flip of the peptide bond Val131−Glu132 and considerable movements of C_α_ atoms of Pro128−Glu132 and the side chains of Arg124, Arg127, Val131, and Glu132 (Fig 3A). Arg127 of Tk-PTP corresponds to a highly conserved arginine residue that plays a key structural role in sustaining the P-loop conformation of DUSP proteins. It mediates hydrogen bonds between its guanidinium group and the main chain carbonyl groups of the fourth and fifth residues of the PTP signature motif in a number of DUSP proteins (S2 Fig) and in Tk-PTP(form II) (Fig 3A, bottom). However, along with the movement of the flexible GG motif, the guanidinium group of Arg127 in Tk-PTP(form I) interacts with the backbone carbonyl group of Leu97 and the side chain hydroxyl group of Ser100, instead of the backbone carbonyl groups of Gly95 and Gly96 (Fig 3A, top). A hydrogen bond between the backbone carbonyl group of the sixth residue (Leu97 in Tk-PTP) of the PTP signature motif and the main chain amide from the α4−α5 loop is also shown in the Tk-PTP(from II) and in a variety of DUSP structures (marked with asterisks in Fig 3A and S2 Fig), but not in the Tk-PTP(form I) structure. Moreover, the guanidinium group of Arg124 in Tk-PTP(form II), but not that in Tk-PTP(form I), forms hydrogen bonds with main chain carbonyl groups of Ala130 and Val131 (Fig 3A). The side chain of Arg124 in the Tk-PTP(form I) structure is in association with residues from a crystal symmetry-related Tk-PTP molecule (S3A Fig). Thus, its conformation observed in the crystalline state might be different from that in solution. Nevertheless, modeling the rotation of the side chain of Arg124 suggests that its guanidinium group is far from main chain carbonyl groups of the α4−α5 loop residues even after being rotated. Therefore it is not expected to form hydrogen bonds with those groups, unlike that in the Tk-PTP(form II) structure (S3B Fig). Collectively, a well-ordered hydrogen bond network is shown in the Tk-PTP(form II) structure (Fig 3A, bottom) like in the various DUSP structures (S2 Fig), in which α4−α5 loop residues (Arg124, Arg127, Ala130, and Val131) and P-loop residues (Met94, Gly95, Leu97, Gly98, and Arg99) are involved, but it is not shown in the Tk-PTP(form I) structure (Fig 3A, top). It is assumed that the temperature-dependent P-loop organization of Tk-PTP is accompanied by conformational change of the neighboring loop, leading to the formation of a hydrogen bond network that might contribute in sustaining the activation loop structure.

### Structural flexibility of Tk-PTP is dependent on the presence of the GG motif

**Fig 4:**
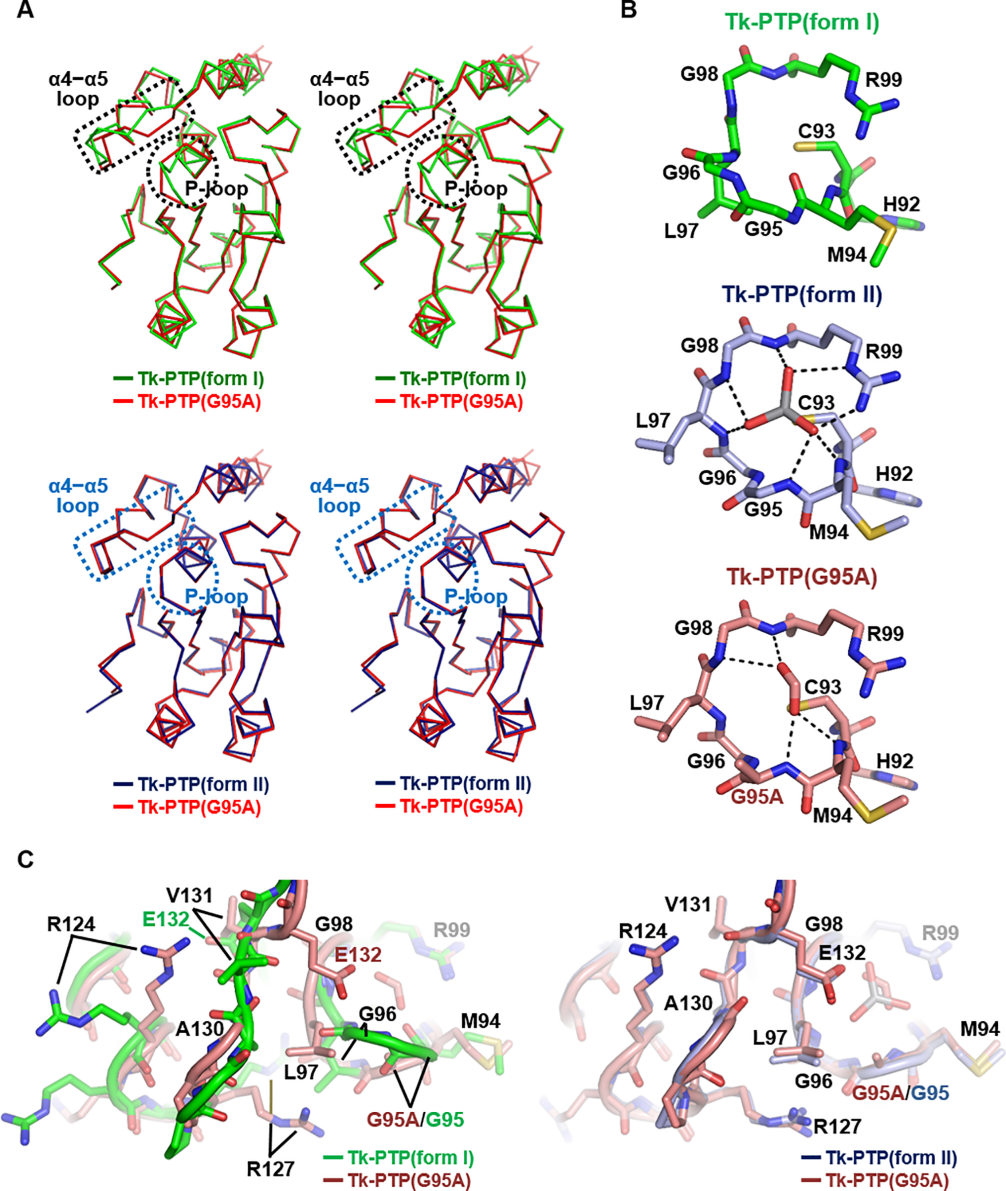
Structural analysis of Tk-PTP(G95A). (A) Stereo views of C_α_ trace of Tk-PTP(G95A) that are superimposed onto that of two forms of Tk-PTP. Dotted circles and rectangles indicate the P-loop and α4−α5 loop regions, respectively. (B) The P-loops of three Tk-PTP proteins are shown as labeled sticks and compared. Dashed lines represent hydrogen bonds and electrostatic interactions mediated by vanadate bound to Tk-PTP(form II) or by formate bound to Tk-PTP(G95A). (C) The P-loop and α4−α5 loop regions of Tk-PTP(G95A) are structurally aligned to those of the two forms of Tk-PTP.

To the best of our knowledge, the described temperature-dependent structural flexibility of Tk-PTP is unique and the first report in the PTP proteins. To confirm whether the unusual structural conversion of Tk-PTP is indeed associated with the presence of a flexible GG motif within its P-loop, we prepared a recombinant Tk-PTP protein containing the Gly95Ala mutation, referred to as Tk-PTP(G95A). The GG motif within the Tk-PTP P-loop was abolished by this mutation; therefore, it was expected that Tk-PTP(G95A) would have a canonically arranged P-loop, similar to that of Tk-PTP(form II). Tk-PTP(G95A) was expressed and purified as was Tk-PTP(form I), and then subjected to crystallization trials without being heated. The structure of Tk-PTP(G95A) was determined up to a resolution of 2.3 Å using cubic-shaped crystals with the space group *H*3 (Table 1). Intriguingly, the resulting crystal structure of Tk-PTP(G95A) was nearly identical to that of Tk-PTP(form II), especially in terms of the conformation of both P-loop and α4−α5 loop (Fig 4A). The P-loop of Tk-PTP(G95A) matched very well with that of Tk-PTP(form II) with RMSDs of 0.16 Å over eight C_α_ atoms and 0.25 Å over the entire 55 atoms. Thus, it adopts a catalytically active P-loop conformation, unlike that of Tk-PTP(form I) (Fig 4B). Accordingly, the ϕ and ψ dihedral angles of the 4^th^ (Ala95) and 5^th^ (Gly96) residues of the Tk-PTP(G95A) P-loop are similar to those of Tk-PTP(form II), but quite different from those of Tk-PTP(form I) (S1 Fig). Taken together, the α4−α5 loop of Tk-PTP(G95A) is structurally similar to the corresponding loop of Tk-PTP(form II) (Fig 4C). Collectively, this structural analysis indicates that Tk-PTP(G95A) adopts an enzymatically active structure even without heat treatment, and that the presence of the unusual GG motif in the P-loop enables the described structural flexibility of wild-type Tk-PTP, the characterizing feature of this protein differentiating it from other PTP proteins.

### Biochemical characterization of phosphatase activity of Tk-PTP

**Fig 5:**
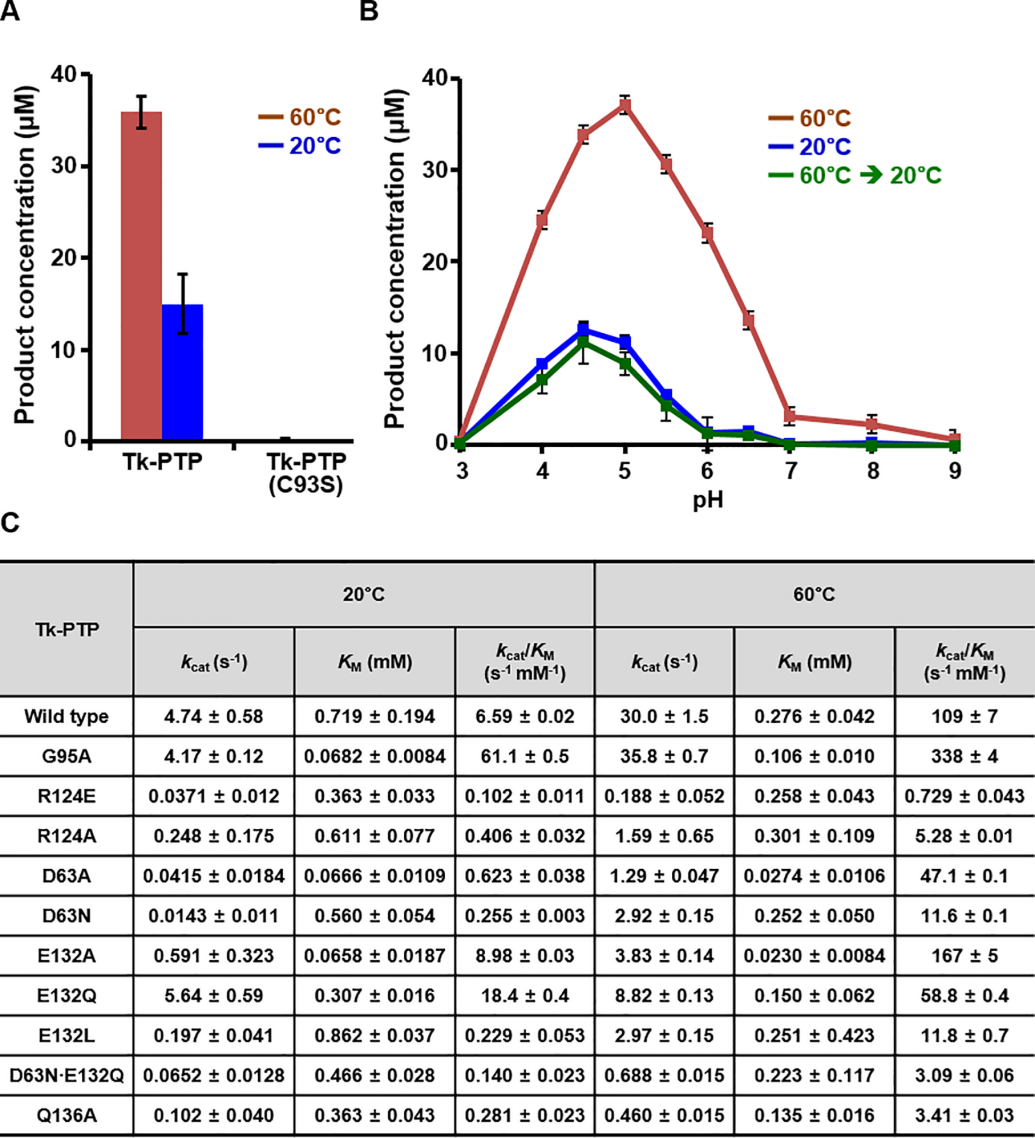
Characterization of enzymatic activity of Tk-PTP. Phosphatase activity assays were carried out at 20°C and 60°C as described in the Materials and Methods section. (A–B) Enzymatic reactions were carried out using 100 μM DiFMUP for 1 h with 10 nM purified recombinant Tk-PTP proteins in the 100 μL reaction buffer at pH 5.0 (*A*) or at the indicated pH levels (*B*). Tk-PTP, but not Tk-PTP(C93S), has dephosphorylating activity (*A*), which is optimum at pH 4.5–5.0 (*B*). (C) Kinetic parameters of 11 types of Tk-PTP proteins are listed. Enzymatic reactions were carried out with purified recombinant Tk-PTP proteins in 100 μL reaction buffer (pH 5.0). Production of DiFMU was detected by measuring fluorescence at 2 min intervals for 10 min, with DiFMUP concentrations of 50, 100, 250, 500, 1000, and 1500 μM. Initial velocity data at each substrate concentration were obtained by detecting the release of DiFMU between 2 and 10 min after the start of reaction, which was calculated from the slope of the each progress curve. Using these data, Michaelis-Menten curves shown in S6 Fig were obtained using the program OriginPro 8.0, by fitting the initial velocities against each DiFMUP concentrations to the hill equation with hill coefficient of 1. Subsequently, Lineweaver-Burk plots shown in S7 Fig were interpreted for the determination of V_max_, *k*_cat_ and *K*_M_ values.

Since the proper conformational arrangement of the P-loop is known to be a critical factor for the catalytic activity of PTP proteins, we analyzed whether the structural conversion of Tk-PTP is accompanied by the regulation of its phosphatase activity. To this end, the dephosphorylating activities of wild-type and a number of mutant recombinant Tk-PTP proteins were measured at 20°C and at 60°C using 6,8-difluoro-4-methylumbelliferyl phosphate (DiFMUP) as a substrate and were compared to each other. We attempted the same assay at 80°C, but were unsuccessful in obtaining reliable kinetic parameters due to excessive production of DiFMU from DiFMUP at that temperature, irrespective of the presence or absence of Tk-PTP (not shown). First, the production of DiFMU was detected in the reaction mixture containing wild-type Tk-PTP, but not Tk-PTP(C93S), demonstrating that Tk-PTP is a cysteine-based enzymatically active phosphatase (Fig 5A). The catalytic activity of Tk-PTP was optimal at pH 4.5−5.0 (Fig 5B), and was clearly higher at 60°C (in which Tk-PTP is presumed to exist as form II) than at 20°C (in which Tk-PTP is presumed to exist as the form I) (Fig 5A and 5B). Critically, the enzymatic activity of Tk-PTP was not affected by pre-heating the protein at 60°C for 3 hour (Fig 5B), indicating that the temperature-dependent structural conversion of Tk-PTP is reversible. Next, we calculated the kinetic constants of recombinant Tk-PTP proteins, which are listed in Fig 5C. The *k*_cat_, *K*_M_, and *k*_cat_/*K*_M_ of wild-type Tk-PTP were calculated to be 4.74 s^-1^, 0.719 mM, and 6.59 s^-1^ mM^-1^ at 20°C and 30.0 s^-1^, 0.276 mM, and 109 s^-1^ mM^-1^ at 60°C (Fig 5C, row 1), showing that the PTP activity of Tk-PTP is 16.5 times higher at 60°C than at 20°C, which is consistent with the structural analysis described in Figs 1–3. The catalytic activities of Tk-PTP(G95A), which constitutively adopts a catalytically active form (see Fig 4), was also measured by the same method. At both temperatures, *k*_cat_ of Tk-PTP(G95A) was similar to that of wild-type Tk-PTP, whereas *K*_M_ of Tk-PTP(G95A) was lower than that of wild-type Tk-PTP, resulting in a much higher *k*_cat_/*K*_M_ ratio for Tk-PTP(G95A) (61.1 s^-1^ mM^-1^ at 20°C and 338 s^-1^ mM^-1^ at 60°C; Fig 5C, row 2) than for wild-type Tk-PTP. These results imply that the phosphatase activity of Tk-PTP can be negatively regulated by the flexibility of the P-loop, which modulates the affinity for substrate, presumably by preventing the binding of substrates to the active site pocket via steric hindrance (see Fig 1D). Two additional mutant proteins were prepared to strengthen the significance of the structural shift between the two forms of Tk-PTP for its enzymatic activity: Tk-PTP(R124E) and Tk-PTP(R124A). As shown in Fig 3A, the side chain of Arg124 plays a key role in sustaining the Tk-PTP(form II) structure by mediating hydrogen bonds with main chain carbonyl groups of Ala130 and Val131. Interestingly, the enzymatic activity of Tk-PTP was greatly impaired by the substitution of this arginine to glutamate or alanine (Fig 5C, rows 3 and 4), demonstrating the significance of intramolecular interactions in maintaining Tk-PTP in the catalytically active form.

### Tk-PTP contains dual general acid/base residues

Along with the catalytic cysteine residue, the PTP proteins commonly share a highly conserved aspartate residue that is critical for enzymatic activity, usually called the general acid/base residue. During the dephosphorylating reaction, this residue provides a side chain carboxylic acid, which first acts as a general acid donating a proton to the Oη atom of the leaving tyrosine residue. In the subsequent steps, it serves as a general base through its deprotonated side chain carboxylate, by activating a water molecule that hydrolyzes the phosphoenzyme intermediate [3]. In most DUSP proteins, an aspartate residue in the β4−α2 loop region is responsible for this function. One exception is DUSP23a, in which Glu134 rather than the highly conserved Asp65 functions as the primary general acid/base residue, which was revealed by the mutant study [27]. In other words, DUSP23a has been considered as the sole DUSP protein that utilizes a glutamate residue as an alternative general acid/base residue, which was revealed by a protein structure-based mutant study [27]. In the previous report by Jeon *et al*, it was suggested that Tk-PTP might also have an alternative general acid/base residue for its enzymatic activity, as its phosphatase activity was not significantly impaired by alanine substitution of the highly conserved aspartate residue, Asp63 [15]. To understand the enzymatic action mechanism of Tk-PTP precisely, one needs to elucidate whether this protein contains additional general acid/base residue(s) substituting the role of Asp63. To resolve this issue based on our structures, we searched for the presence of acidic residues directing the active site pocket of Tk-PTP. As shown in Fig 3B, Asp63 of Tk-PTP was observed to be definitely oriented towards the catalytic pocket in both the structures, as were the corresponding aspartate residues in three other representative DUSP structures (S4 Fig), strongly suggesting that Asp63 is able to function as the typical general acid/base residue in Tk-PTP. Intriguingly, another putative general acid/base residue was also found in Tk-PTP. While the side chain of Glu132 is directed away from the catalytic pocket in Tk-PTP(form I), it exhibits a drastic conformational change and is reoriented to the catalytic center in Tk-PTP(form II). This relocation is accompanied by a flip of the peptide bond Val131−Glu132 (Fig 3B). It also alters the ψ angle of Glu132 from 140.22° in Tk-PTP(form I) to -25.51° in Tk-PTP(form II), which is consistent with the molecular dynamics simulation showing the temperature-dependent frequency shift of the dihedral angles of Glu132 (S5 Fig). Glu132 in Tk-PTP(form II) is structurally matched to Gln446 in YopH (S4 Fig) which is known to be the Q-loop glutamine residue coordinating a water molecule necessary for hydrolysis during the dephosphorylation reaction [4]. This glutamine is highly conserved in a wide variety of receptor type and non-receptor type PTP proteins, but not in DUSP proteins (S4 Fig). Instead, Glu134 of DUSP23a, the uniquely identified alternative general acid/base residue found in DUSP proteins [27], is located in this position (S4 Fig), implying that Glu132 of Tk-PTP also plays a similar role in the dephosphorylating reaction as the second general acid/base residue.

Next, to verify the functional significance of Asp63 and Glu132 and their weight of contribution to the enzymatic activity, a number of mutant recombinant Tk-PTP proteins were prepared and subjected to enzyme activity assays. Since alanine substitution of Asp63 or Glu132 caused a decrease of *K*_M_ values that might be due to unintendedly enhanced substrate accessibility (Fig 5C, rows 5 and 7), we introduced Asp-to-Asn and Glu-to-Gln/Leu substitution(s) into Tk-PTP and assayed the catalytic activities of the resulting recombinant proteins. First, introduction of the D63N mutation remarkably attenuated the enzymatic activity of Tk-PTP, with the reduction of *k*_cat_/*K*_M_ values by one order of magnitude at both 20°C and 60°C (Fig 5C, row 6). In contrast, glutamine substitution of Glu132 resulted in relatively moderate effect on the phosphatase activity of Tk-PTP; *k*_cat_/*K*_M_ of Tk-PTP was rather increased at 20°C and decreased only in half at 60°C by the introduction of the E132Q mutation (Fig 5C, row 8). This might be because the mutation led to the creation of an artificial Q-loop with the introduced glutamine, which unexpectedly assisted the dephosphorylating activity of the mutant protein. Thus, a mutant Tk-PTP protein containing a E132L mutation was prepared, which showed markedly reduced phosphatase activity (Fig 5C, row 9) to a degree similar to that of Tk-PTP(D63N). We note that the relatively low phosphatase activity of Tk-PTP at 20°C compared to that at 60°C was further attenuated not only by the D63N mutation but also by the E132L substitution, suggesting a possibility that the structural relocation of Glu132 might take place also at low temperature during enzyme reaction, but at much lower rate compared to that at high temperature. Furthermore, double mutation of Asp63 and Glu132 to asparagine and glutamine, respectively, abrogated the enzymatic activity of Tk-PTP synergistically (Fig 5C, row 10). These results collectively demonstrated that Asp63 and Glu132 cooperate as dual general acid/base residues in the dephosphorylating reaction catalyzed by Tk-PTP. Furthermore, these results also supported the significance of the conformational change of Tk-PTP for phosphatase reactivity, as Glu132 of Tk-PTP is able to work as the general acid/base residue only in Tk-PTP(form II) but not in Tk-PTP(form I). Finally, since Gln138 of DUSP23a was previously proven to play a critical role in the enzymatic reaction by stabilizing the two acidic residues through water-mediated hydrogen bonds [27], Tk-PTP(Q136A) was also prepared and subjected to the enzymatic assay. This mutant protein showed severely impaired phosphatase activity (Fig 5C, row 11), indicating the importance of the Gln136-mediated hydrogen bond network in Tk-PTP that structurally supports the coordination of the two acidic residues (Fig 3B).

### Hyperthermostability of Tk-PTP depends on its intramolecular hydrophobic interactions

**Fig 6:**
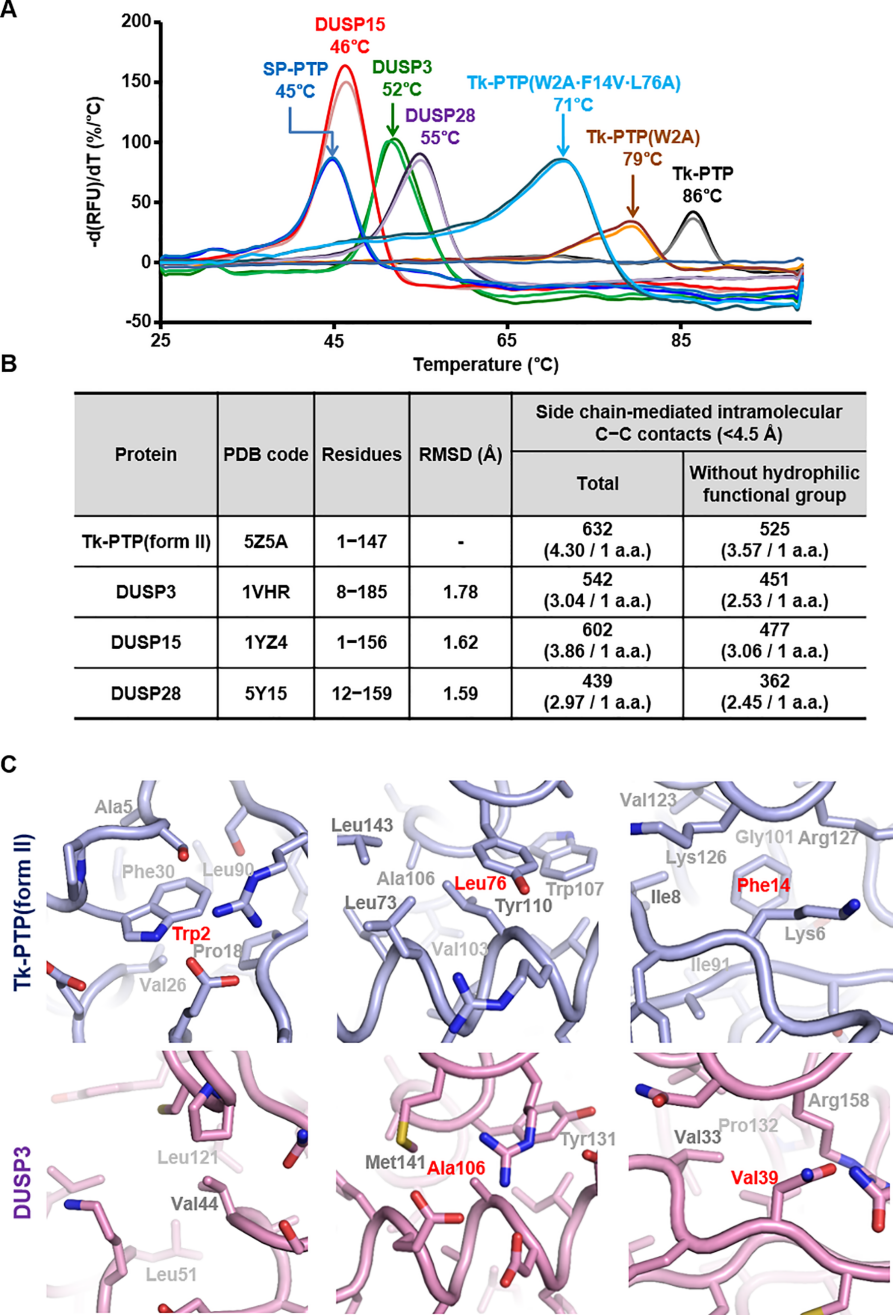
Characterization of thermostability of Tk-PTP. (A) Melting points of three different constructs of Tk-PTP and four PTP proteins. All the measurements were carried out twice. (B) Number of intramolecular carbon−carbon contacts within 4.5 Å mediated by side chain atoms of Tk-PTP and three human DUSP proteins are analyzed and compared. “Hydrophilic functional group” includes imidazole of histidine, guanidinium of arginine, carboxylates of aspartate or glutamate, and amides of asparagine or glutamine. (C) Intramolecular interactions within Tk-PTP(form II) and DUSP3. The three hydrophobic residues of Tk-PTP that were substituted in *A* and the corresponding residues of DUSP3 are marked in red.

*T*. *kodakaraensis* KOD1 is a solfatara-isolated hyperthermophilic archaeon, and the enhanced enzymatic activity of Tk-PTP at 60°C compared to the activity at 20°C indicates that this protein is stable under high temperature, unlike other DUSP proteins. To confirm the thermostability of Tk-PTP, the melting temperatures (T_m_) of Tk-PTP and those of four other PTPs were measured and compared. As shown in Fig 6A, the T_m_ of Tk-PTP was determined to be 86°C, which is clearly much higher than those of DUSP3 (52°C), DUSP15 (46°C), DUSP28 (55°C), and SP-PTP (45°C), a bacterial PTP protein from *Streptococcus pyogenes* [28,29] (Fig 5A). Since the backbone fold of Tk-PTP did not show a notable difference when compared to that of the human DUSP proteins (see Fig 1B), we hypothesized that the high structural rigidity of Tk-PTP might have resulted from the interior composition of side chains that are expected to be tightly packed. As shown in Fig 6B, the interior of Tk-PTP is filled with tighter intermolecular carbon−carbon contacts compared to those of the other three DUSP proteins, despite their overall structural similarity. To verify the contribution of internal carbon−carbon contacts to the thermostability of Tk-PTP, two mutant Tk-PTP proteins were prepared. Their phosphatase activities were mostly retained, and therefore the overall structures of these mutant proteins are presumed to be maintained (S8 Fig). First, alanine substitution of nonconserved Trp2 of Tk-PTP, whose side chain is involved in 36 intramolecular carbon−carbon contacts within 4.5 Å (Fig 6C, left), reduced the T_m_ value by 7°C (Fig 6A). Two additional residues, Phe14 and Leu76, were selected for further mutational study based on the structural comparison between Tk-PTP and DUSP3 (Fig 6C, middle and right). The T_m_ further dropped to 71°C by additionally introducing F14V and L76A substitutions (Fig 6A), as the number of the carbon−carbon contacts were modeled to decrease from 34 (Phe14 or Leu76-mediated) to 11 (Val14 or Ala76-mediated). Collectively, these data indicate that the hyperthermostability of Tk-PTP arises from its tightly packed protein interiors, mostly based on the intramolecular hydrophobic interactions.

### Structural comparison with SsoPTP

**Fig 7:**
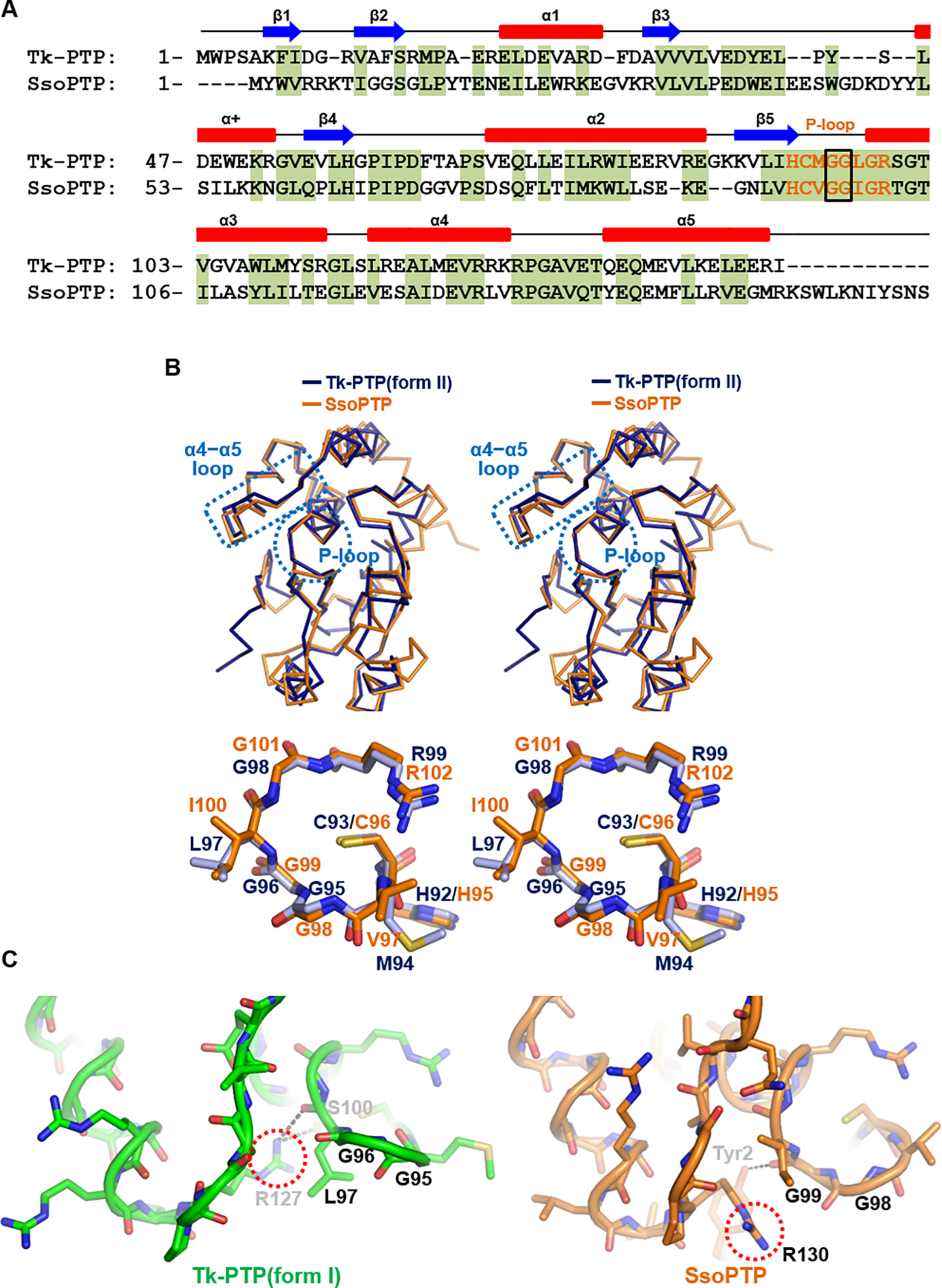
Structural comparison with SsoPTP. (A) Sequence alignment of Tk-PTP and SsoPTP. The secondary structure of Tk-PTP is shown together. Aligned residues are shaded green. The P-loop residues are presented in orange, and the GG motifs are highlighted by rectangles. (B) Structural alignment of Tk-PTP(form II) and SsoPTP in stereo views. C_α_ trace (top) and P-loop in sticks (bottom) of Tk-PTP(form II) are superimposed on those of SsoPTP. The P-loop and α4−α5 loop regions are indicated by dotted circles or rectangles, respectively (*top*). (C) The P-loop and α4−α5 loop regions of Tk-PTP(form II) are structurally compared to those of SsoPTP. Dotted circles highlight the guanidinium group of Arg127 of Tk-PTP(form II) or that of Arg130 of SsoPTP. Hydrogen bonds described in the main text are presented as dashed lines.

As mentioned previously, SsoPTP from the thermophilic archaeon *Sulfolobus solfataricus* was the only archaeal PTP protein whose structure was determined in apo or phosphate/tungstate/para-nitrophenylphosphate/phosphopeptide-bound forms [12]. Even though *S*. *solfataricus* and *T*. *kodakarensis* belong to different archaea kingdoms (crenarchaeota and euryarchaeota, respectively), SsoPTP and Tk-PTP share considerable sequence homology (31% identity and 50% similarity; Fig 7A). Furthermore, the two proteins commonly contain the GG motif in their P-loop region (Fig 7A) that is absent in 28 DUSP proteins (see Fig 2F). Consistently, a DALI search with Tk-PTP(form I) identified SsoPTP as a homologue with the Z-score 20.3. Nonetheless, a critical difference between the two archaeal PTP proteins was found. Unlike the crystal structures of Tk-PTP, all the crystal structures of SsoPTP adopt only a typical active DUSP form, even without being treated with heat during protein purification [12], suggesting that SsoPTP might not undergo a structural change between different forms. When superimposed, the overall structure (an RMSD of 1.27 Å over 136 aligned residues; Fig 7B, top) and the conformation of the P-loop (Fig 7B, bottom) of SsoPTP and Tk-PTP(form II) aligned well with each other, demonstrating the structural similarity between the two archaeal PTP proteins as the catalytically active form. Next, in order to elucidate why SsoPTP does not adopt an enzymatically inactive form unlike Tk-PTP, despite the presence of the GG motif in its P-loop region, we structurally aligned and compared the crystal structures of SsoPTP and Tk-PTP(form I). In Tk-PTP(form I), the guanidinium group of Arg127 is directed to the interior of the protein and interacts with the P-loop residue atoms including the main chain carbonyl group of Leu97 and the side chain hydroxyl group of Ser100, presumably anchoring the P-loop of Tk-PTP(form I) in the atypical conformation (Fig 7C, left). However, the presence of nonconserved Tyr2 in SsoPTP prevents Arg130 from being directed to the interior of the protein (Fig 7C, right). Furthermore, the hydroxyl group of Tyr2 forms a hydrogen bond with the main chain carbonyl group of Gly99, which should stabilize the P-loop of SsoPTP in the canonical conformation (Fig 7C, right). This structural analysis implies that the presence of the GG motif is necessary but not sufficient for the structural flexibility of archaeal PTP proteins. A BLAST search for homologous proteins using the amino acid sequence of Tk-PTP revealed a diversity of putative PTP proteins originating from archaeal kingdoms euryarchaeota, crenarchaeota, and thaumarchaeota (S9 Fig) The GG motif in the P-loop is conserved in most PTP proteins from *Thermococcus*, *Pyrococcus*, *Palaeococcus*, *Sulfolobus*, *Staphylothermus*, and so on, but not in proteins from *Vulcanisaeta*, *Candidatus*, or *Halococcus* (S9 Fig). Whether the presence of the GG motif causes conformational flexibility in those archaeal proteins as in Tk-PTP or not (as in SsoPTP) remains to be determined by structural and biochemical elucidation.

## Discussion

In this work, we presented in-depth structural and biochemical analyses based on the newly determined crystal structures of three forms of Tk-PTP, the PTP protein from *T*. *kodakaraensis* KOD1. The overall structures of all the three forms of Tk-PTP are highly homologous to the common DUSP proteins, composed of a central β-sheet sandwiched by α-helices on both sides, which is consistent with a previous study reporting that Tk-PTP dephosphorylates both phsophotyrosine and phosphoserine [15]. Nevertheless, Tk-PTP was also revealed to have its own unique structural features differentiating this protein from any other PTP/DUSP proteins. The T_m_ of Tk-PTP was measured to be much higher than that of human DUSP proteins (Fig 6A), and its hyperthermostability was proven to be mediated by the tight intramolecular hydrophobic interactions (Fig 6B and 6C). Furthermore, the fourth (Gly95) and fifth (Gly96) residues of the PTP consensus motif of Tk-PTP constitute a GG motif, which provides structural flexibility to the P-loop of Tk-PTP, accompanied by the conformational change of the P-loop-contacting α4−α5 region (Figs 2 and 3). Even though a similar conformational change is found in the MKP subfamily of DUSP proteins, it requires the MKB domain-mediated protein−protein interaction [22–26]. In contrast, Tk-PTP is composed of a single catalytic domain without any accessory domain; therefore, its structural switch, which appears to occur by itself in a temperature-dependent manner without any help of a binding partner, was wholly unexpected before structure determination. Despite that temperature-dependent conformational changes have been reported and structurally analyzed in a number of proteins [30–32], to the best of our knowledge, this is a novel and unique report in the PTP proteins. We consider that such a conformational switch would significantly affect the enzymatic activity of Tk-PTP. Thus, it might be a novel mechanism for the regulation of its phosphatase activity because of the following reasons. First, steric hindrance occurs between a phosphate group and the P-loop of Tk-PTP(form I) (Fig 1D). Second, the P-loop of Tk-PTP adopts a canonical conformation in Tk-PTP(form II) but not in Tk-PTP(form I) (Fig 2E). Third, the introduction of G95A substitution that restricts the flexibility of the P-loop (Fig 4) led to a nine-fold improvement in enzymatic activity at 20°C and three-fold improvement at 60°C (Fig 5C). These results collectively indicate that the dephosphorylating capacity of the two forms are quite different from each other, and that the enhanced *k*_cat_/*K*_M_ value of Tk-PTP at 60°C compared to the one at 20°C is not only due to thermodynamics, but also due to a conformational switch that controls its enzymatic activity.

Thus far, DUSP23a was the sole PTP known to contain dual general acid/base residues [27]. Through structural elucidation, we found the presence of the second general acid/base residue (Glu132; Fig 3B) in Tk-PTP, as predicted by Jeon *et al*. [15]. The leucine substitution of Glu132 resulted in the attenuation of dephosphorylating activity of Tk-PTP, 28.8 times at 20°C and 9.24 times at 60°C (Fig 5C, row 9), suggesting the significance of this residue for the enzymatic activity of Tk-PTP. Nevertheless, contrary to the previous report by Jeon *et al*., which noted that alanine substitution of Asp63 in Tk-PTP did not impair its enzymatic activity [15], our structural and biochemical analysis demonstrated that the highly conserved canonical general acid/base residue Asp63 in Tk-PTP also plays a critical role in its dephosphorylating reaction (Figs 3B and 5C). We suppose that alanine substitution of a general acid/base residue(s) might not be the best option in biochemical assays using PTP proteins, as it caused unintended change in the *K*_m_ value due to the enhanced substrate accessibility, as presented in this study (Fig 5C, rows 5 and 7) and as shown by others [27]. Moreover, we found that catalytic parameters were not accurately obtained from the biochemical assays carried out at 80°C at least under our experimental conditions because of the uncontrolled degradation of the substrate molecule. As the asparagine substitution of Asp63 impaired the phosphatase activity of Tk-PTP to nearly the same degree as the leucine substitution of Glu132 did (Fig 5C, rows 6 and 9), we concluded that both amino acids function as general acid/base residues during the reaction, and are crucial for the phosphatase activity of this protein. We also note that Glu132 orients towards the catalytic pocket only in Tk-PTP(form II) but not in Tk-PTP(form I) (Fig 3B), further supporting the association of the conformational change with the enzymatic activity of Tk-PTP.

An important issue that remains is the biological benefit that the archaeon receives from such structural flexibility of Tk-PTP, which appears to lower its enzymatic activity. The GG motif necessary for the conformational switch of Tk-PTP is not conserved in DUSP proteins (Fig 2F) except for those from thermoarchaea (S9 Fig), suggesting that the structural flexibility shown in Tk-PTP might be somehow advantageous to archaea inhabiting high temperature environments. *T*. *kodakaraensis* KOD1, a representative thermophilic archaea, is known to grow at temperatures ranging from 60°C to 100°C [13], implying that its biological processes would be stopped or attenuated when this archaeon is exposed to low temperature conditions. One possibility is that such a “shutdown” might take place not passively but under control, and that the conformational switch of Tk-PTP to the inactive form might provide a route to stop the intracellular signaling pathway of this archaeon by blocking dephosphorylation mediated by this protein. Another possibility is that the conformational change of Tk-PTP might be induced by other unknown environmental factors or by unresolved protein−protein interactions via the catalytic domain. We consider that further verification of the precise function and significance of the phosphatase activity of Tk-PTP in the physiology of *T*. *kodakaraensis* KOD1 and identification of its substrates or binding proteins might help in elucidating this issue.

In this work, we delineated novel and unique structural features of Tk-PTP, which includes temperature-dependent conformational flexibility, a noncanonical motif in the P-loop, dual general acid/base residues, and hyperthermostability. We believe that our structural and biochemical information will be a rational basis for future investigations to expand our understanding of the PTP proteins from the first eukaryotic and the second prokaryotic domains to the third archaeal domain of life.

## Materials and methods

### Preparation, crystallization, and structural determination of three forms of Tk-PTP

The DNA fragment encoding Tk-PTP was amplified by polymerase chain reaction using the *E*. *coli* codon-optimized Tk-PTP gene purchased from Bioneer as a template, and cloned into the pET28a plasmid (Novagen). This construct was used as the template for the preparation of mutant Tk-PTP containing a G95A substitution, which was cloned into the pET21a plasmid (Novagen). Wild type or mutant Tk-PTP protein was produced in the *E*. *coli* BL21(DE3) RIL strain (Novagen) at 25°C and initially purified using a Ni-NTA column (QIAGEN). After the removal of the N-terminal (His)_6_-tag from wild type Tk-PTP through thrombin protease treatment, each protein was further purified using a HiLoad 26/600 Superdex 75 pg gel filtration column (GE Healthcare) equilibrated with a buffer solution containing 50 mM Tris-HCl (pH 7.5), 200 mM NaCl, and 2 mM dithiothreitol. Tk-PTP(form I) crystals were obtained by the sitting-drop vapor diffusion method at 18°C, by mixing and equilibrating a 1 μL protein solution (10 mg/mL) and a 1 μL precipitant solution containing 0.1 M sodium citrate (pH 5.4), 8% (w/v) polyethylene glycol 10000, and 14% (v/v) dioxane. Tk-PTP(G95A) crystals were obtained by the sitting-drop vapor diffusion method at 18°C by mixing and equilibrating a 1 μL protein solution (10 mg/mL) and a 1 μL precipitant solution containing 0.1 M Bis-Tris (pH 6.25), and 0.8 M magnesium formate dehydrate. As mentioned in the main text, a portion of purified protein sample of wild type Tk-PTP was mixed in a 1:3 molar ratio with sodium vanadate (Sigma), and heated at 60°C for 3 hours. Tk-PTP(form II) crystals were obtained using this sample, by the sitting-drop vapor diffusion method at 18°C by mixing and equilibrating a 1 μL protein solution (10 mg/mL) and a 1 μL precipitant solution containing 0.03 M citric acid, 0.07 M Bis-Tris propane (pH 7.6), 8% (w/v) polyethylene glycol 3350, and 0.08 M spermine tetrahydrochloride. Before the data collection process, crystals were immersed briefly in a cryoprotectant solution, which was a reservoir solution containing 20% glycerol for Tk-PTP(form I) and Tk-PTP(form II) and 20% ethylene glycol for Tk-PTP(G95A). Diffraction data were collected on the beamlines 5C and 7A at the Pohang Accelerator Laboratory, Korea, and processed using the program *HKL* 2000 [33]. The structure of Tk-PTP(form I) was determined by molecular replacement method with the program Phaser [34], using the structure of SsoPTP as a search model. The structures of Tk-PTP(form II) and Tk-PTP(G95A) were determined in the same way, using the Tk-PTP(form I) structure as a search model. The programs Coot [35] and PHENIX [36] were used for model building and refinement, respectively. Crystallographic data statistics are summarized in Table 1.

### Preparation of recombinant mutant Tk-PTP proteins and other PTP proteins

Each of the DNA fragments encoding mutant Tk-PTP proteins containing C93S, R124E, R124A, D63A, D63N, E132A, E132Q, E132L, D63N·E132Q, Q136A, W2A, or W2A·F14V·L76A substitution were cloned into the pET21a plasmid. The proteins were produced in the *E*. *coli* BL21(DE3) RIL strain at 25°C and purified using a Ni-NTA column. SP-PTP and three human DUSP proteins, DUSP3, DUSP15, and DUSP28, were prepared and purified as previously described [29,37].

### Phosphatase activity assays

Dephosphorylating activity assays using DiFMUP as a substrate were conducted using purified recombinant Tk-PTP proteins in a 100 μL reaction mixture containing 200 mM NaCl, 5 mM dithiothreitol, and a 100 mM buffer for adjusting pH: to 3.0, 4.0, 4.5, 5.0, and 5.5 with sodium citrate; to 6.0 and 6.5 with MES; to 7.0, 8.0, and 9.0 with Tris-HCl. Enzymatic activities were measured in triplicate by detecting the DiFMU concentration by monitoring the excitation to emission ratio at 355/450 nm on the multimode plate reader Victor X3 (Perkin Elmer).

### Thermal shift assays

Each protein sample was diluted to 0.625 mg/mL in a 20 μL reaction buffer, containing 50 mM Tris-HCl (pH 7.5), 200 mM NaCl, 2 mM dithiothreitol, and 8x diluted SYPRO orange fluorescent dye (Applied Biosystems). Samples were pipetted into 8-well PCR tubes and sealed with optical flat strips (Bio-Rad). All experiments were performed using a Bio-Rad CFX96 real-time system, and the ROX reporter was chosen to collect fluorescent emission signals. Temperature was held for 2 min at the starting (25°C) and the ending (99°C) points, and for 5 sec at 0.5-degree intervals between the starting and the ending points. All samples were run in duplicate.

### Molecular dynamics simulation

Simulations were carried out using Chemistry at HARvard Macromolecular Mechanics (CHARMM) with CHARMM22 force field [38,39] and EEF1.1 implicit solvation model [40]. After steepest descent energy minimization for 50 steps and Newton-Rhapson energy minimization for 50 steps, the Langevin dynamics was performed for 10 ns. The bond lengths of hydrogen atom were fixed via SHAKE. To change the temperature condition, temperature factor used in the Langevin dynamics was increased from 15°C to 95°C in 20°C increments, and a total of nine simulations were performed. The coordinates were saved every 10 ps for analysis. After the dynamics simulation, additional steepest descent and Newton-Rhapson energy minimization were performed at 100 and 3000 steps, respectively. One thousand coordinates were finally obtained from the DCD file through trajectory analysis. Phi and psi dihedral angles of those structures were measured using the PROSS program [41]. After measuring the dihedral angles, the frequency was measured at 10 intervals from -180 to 180 degrees and normalized. The dihedral angle change was observed through the frequency distribution.

### Accession numbers

The coordinates of Tk-PTP(form I), Tk-PTP(form II), and Tk-PTP(G95A) together with the structure factors have been deposited in the Protein Data Bank with the accession codes of 5Z59, 5Z5A, and 5Z5B, respectively.

## Supporting information

S1 Fig ϕ and ψ dihedral angles of the GG motif residues of three forms of Tk-PTP. Dihedral angles 95^th^ (in blue) and 96^th^ (in black) residues of three forms of Tk-PTP are presented on the Ramachandran plot for glycine (*left*) or in the table (*right*).
(TIF)

S2 Fig P-loop and α4−α5 loop regions of three human PTP proteins. Hydrogen bonds between the P-loop and α4−α5 loop residues are shown with dashed lines. The PDB codes for DUSP3, DUSP10, and DUSP23b are 1VHR, 1ZZW, and 3RGQ, respectively.
(TIF)

S3 Fig Conformation of Arg124 in two forms of Tk-PTP. (A) Crystal packing interactions of Arg124 with Glu138 and Glu145 from the neighboring molecule in the Tk-PTP(form I) structure.
(B) Arg124 in the Tk-PTP(form II) structure interacts with the main chain carbonyl groups of α4−α5 loop, whereas the same residue in the Tk-PTP(form I) structure is unable to do that, even in the model where the side chain of Arg124 is rotated.
(TIF)

S4 Fig General acid/base residues of PTP proteins. The conformation of general acid/base residues labeled in red are structurally compared among four PTP structures. The catalytic residues are labeled in gray, and the Q-loop glutamine residue of YopH is labeled in black. Water-mediated hydrogen bonds involving the conformation of general acid/base residues or the Q-loop glutamine residue are presented as dotted lines. The PDB code for DUSP23a and YopH is 4ERC and 1LYV, respectively.
(TIF)

S5 Fig Frequency distribution of dihedral angles from molecular dynamics simulation. The ϕ and ψ values of Glu132 of Tk-PTP obtained from molecular dynamics simulation are shown as graphs. Dihedral angles from the experimentally determined structures are indicated by red and black arrows.
(TIF)

S6 Fig Michaelis-Menten curves of 11 Tk-PTP proteins. (TIF)

S7 Fig Lineweaver-Burk plots of 11 Tk-PTP proteins. Resulting kinetic parameters including *k*_cat_, *K*_M_, and *k*_cat_/*K*_M_ values of those proteins listed in Fig 5C.
(TIF)

S8 Fig Enzymatic activity measurement of two mutant Tk-PTP proteins. Phosphatase activity assays were carried out at 60°C using wild-type and two mutant Tk-PTP proteins in the same way with those in Fig 5A.
(TIF)

S9 Fig P-loop amino acid sequences of putative archaeal PTP proteins. The P-loop residue sequences of 39 putative PTP proteins from euryarchaeota, 19 proteins from crenarchaeota, and 6 proteins from thaumarchaeota kingdoms are aligned. The 4^th^ and 5^th^ residues are marked in red for glycine or in cyan for other residues.
(TIF)
