# Supplementary figures and images for: Structural study reveals the temperature-dependent conformational flexibility of Tk-PTP, a protein tyrosine phosphatase from Thermococcus kodakaraensis KOD1
Source: PLoS One. 2018 May 23;13(5):e0197635. doi: 10.1371/journal.pone.0197635 (PMC5965843; doi:10.1371/journal.pone.0197635)

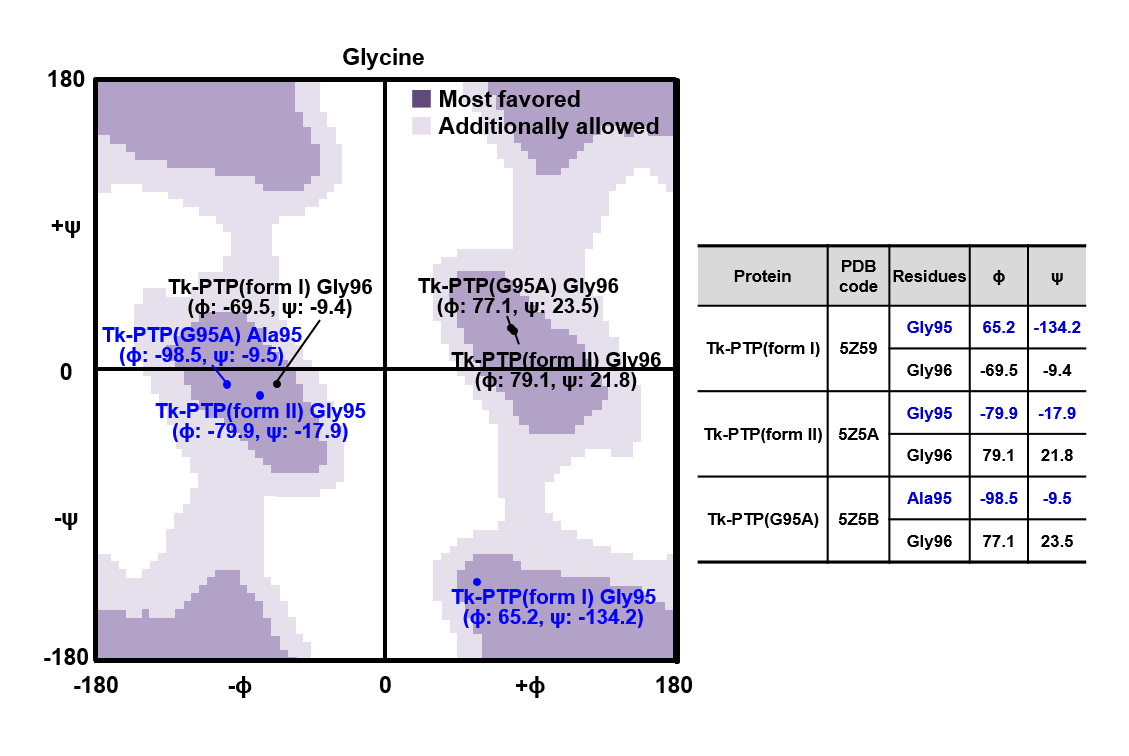

Supplement: S1 Fig — Dihedral angles 95th (in blue) and 96th (in black) residues of three forms of Tk-PTP are presented on the Ramachandran plot for glycine (left) or in the table (right). (TIF) [file pone.0197635.s001.tif]

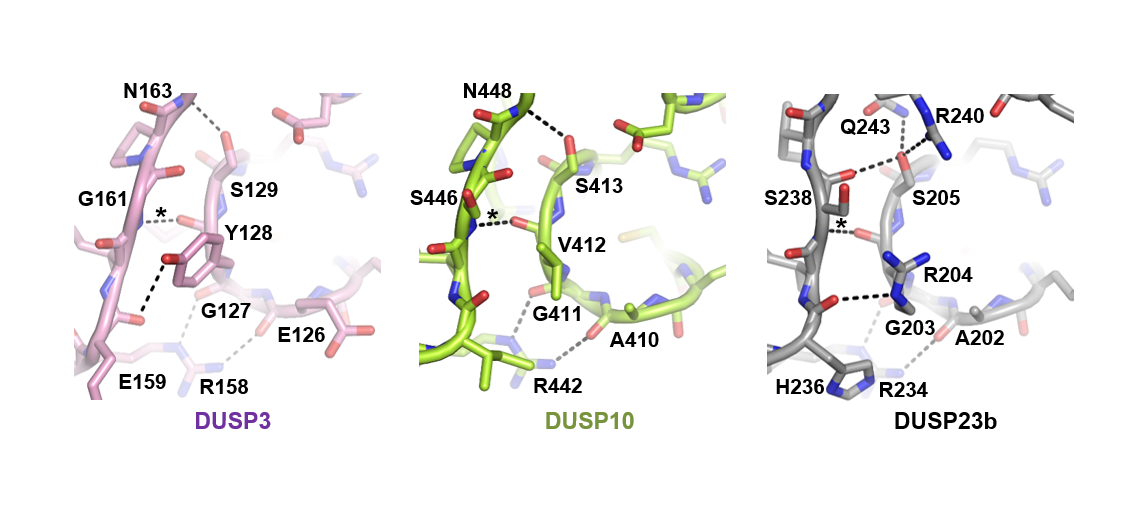

Supplement: S2 Fig — Hydrogen bonds between the P-loop and α4−α5 loop residues are shown with dashed lines. The PDB codes for DUSP3, DUSP10, and DUSP23b are 1VHR, 1ZZW, and 3RGQ, respectively. (TIF) [file pone.0197635.s002.tif]

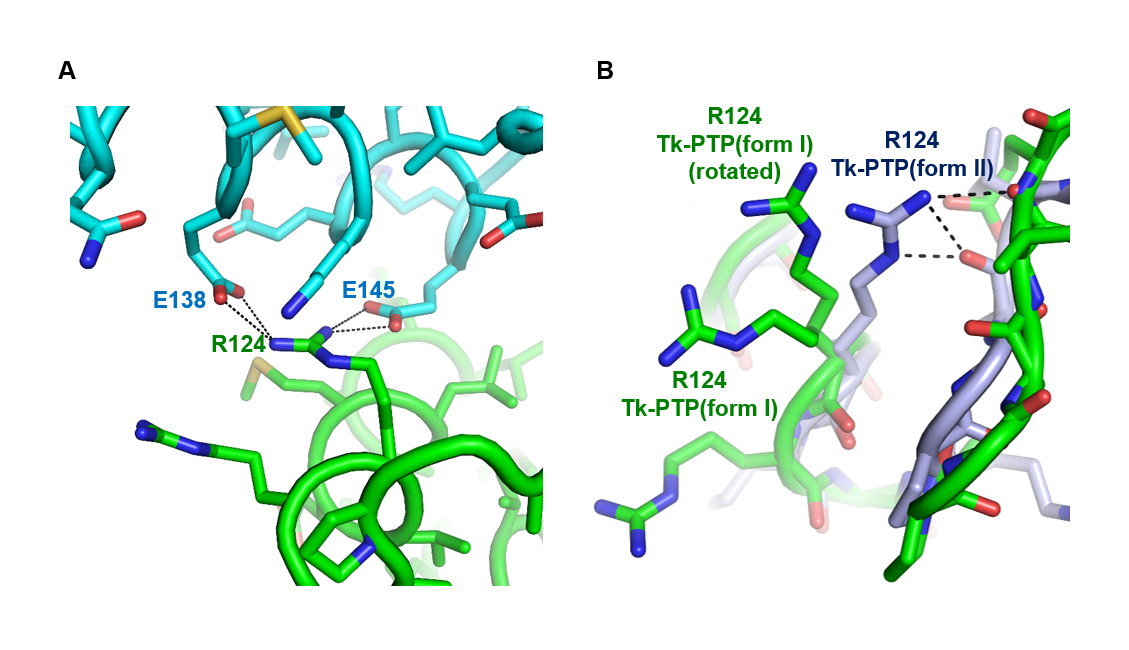

Supplement: S3 Fig — (A) Crystal packing interactions of Arg124 with Glu138 and Glu145 from the neighboring molecule in the Tk-PTP(form I) structure. (B) Arg124 in the Tk-PTP(form II) structure interacts with the main chain carbonyl groups of α4−α5 loop, whereas the same residue in the Tk-PTP(form I) structure is unable to do that, even in the model where the side chain of Arg124 is rotated. (TIF) [file pone.0197635.s003.tif]

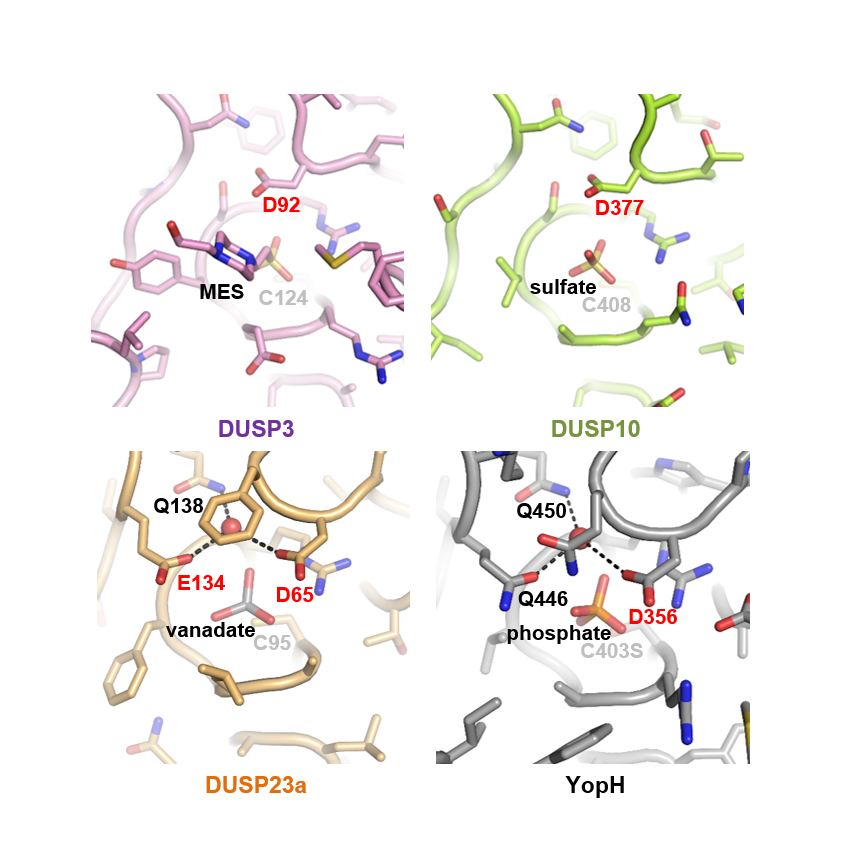

Supplement: S4 Fig — The conformation of general acid/base residues labeled in red are structurally compared among four PTP structures. The catalytic residues are labeled in gray, and the Q-loop glutamine residue of YopH is labeled in black. Water-mediated hydrogen bonds involving the conformation of general acid/base residues or the Q-loop glutamine residue are presented as dotted lines. The PDB code for DUSP23a and YopH is 4ERC and 1LYV, respectively. (TIF) [file pone.0197635.s004.tif]

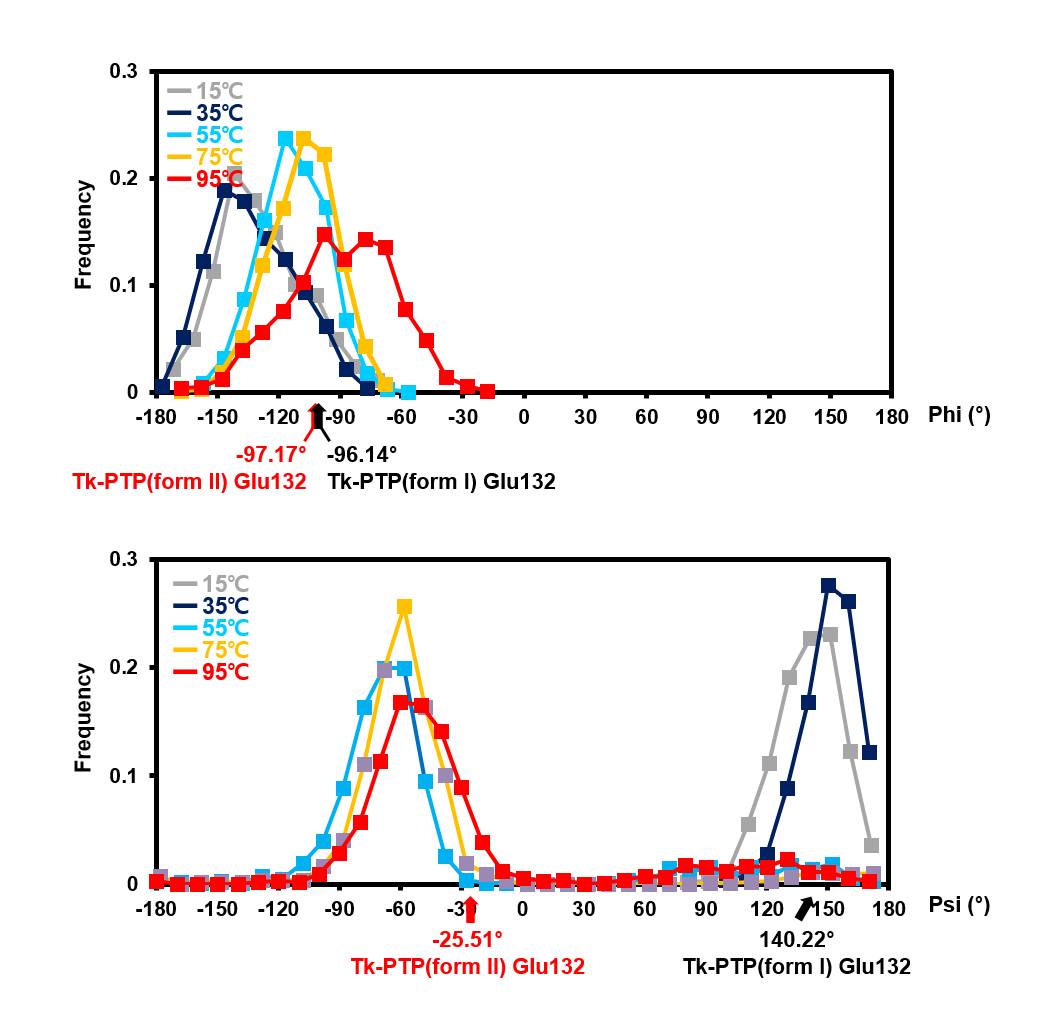

Supplement: S5 Fig — The ϕ and ψ values of Glu132 of Tk-PTP obtained from molecular dynamics simulation are shown as graphs. Dihedral angles from the experimentally determined structures are indicated by red and black arrows. (TIF) [file pone.0197635.s005.tif]

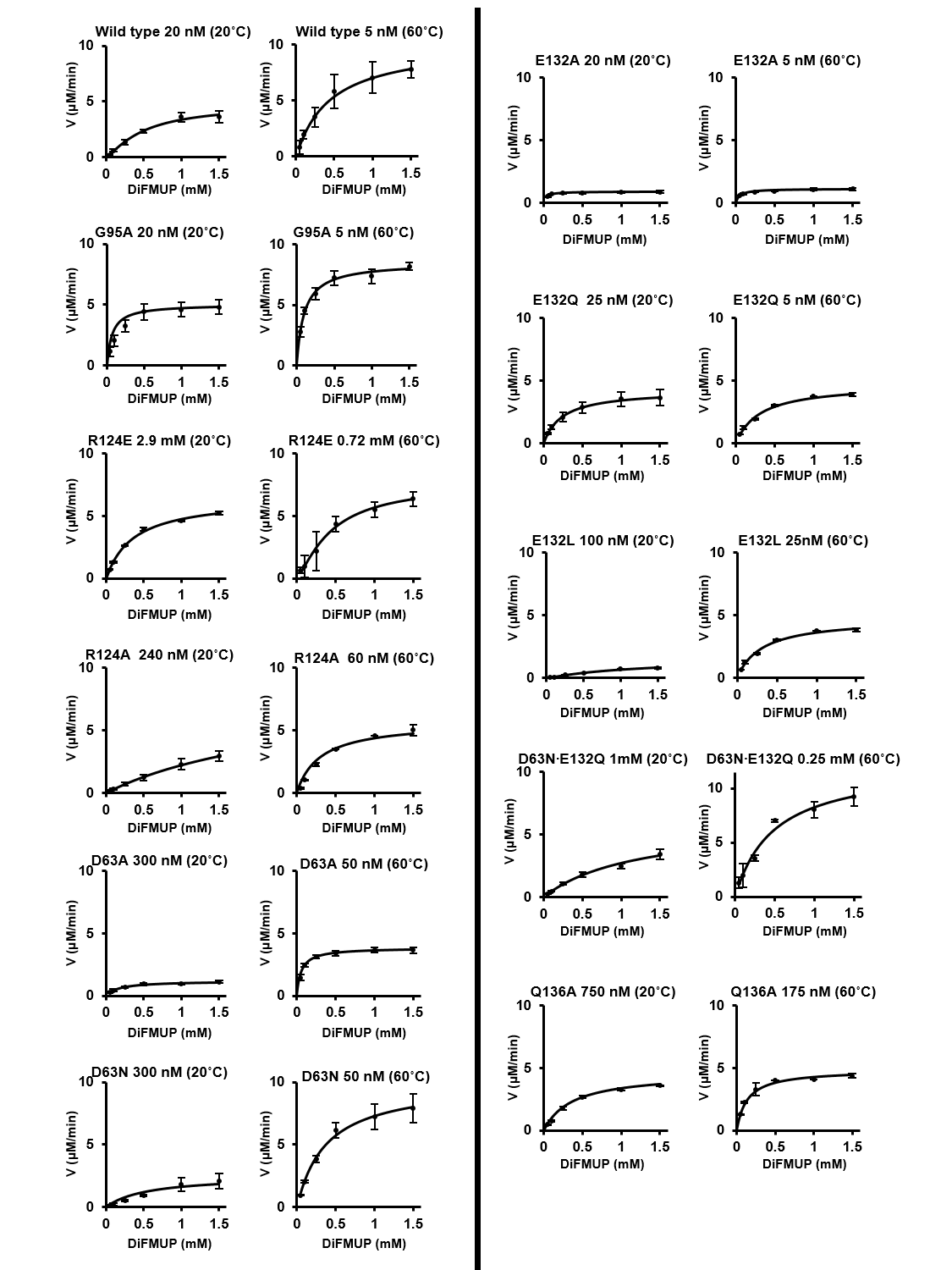

Supplement: S6 Fig — (TIF) [file pone.0197635.s006.tif]

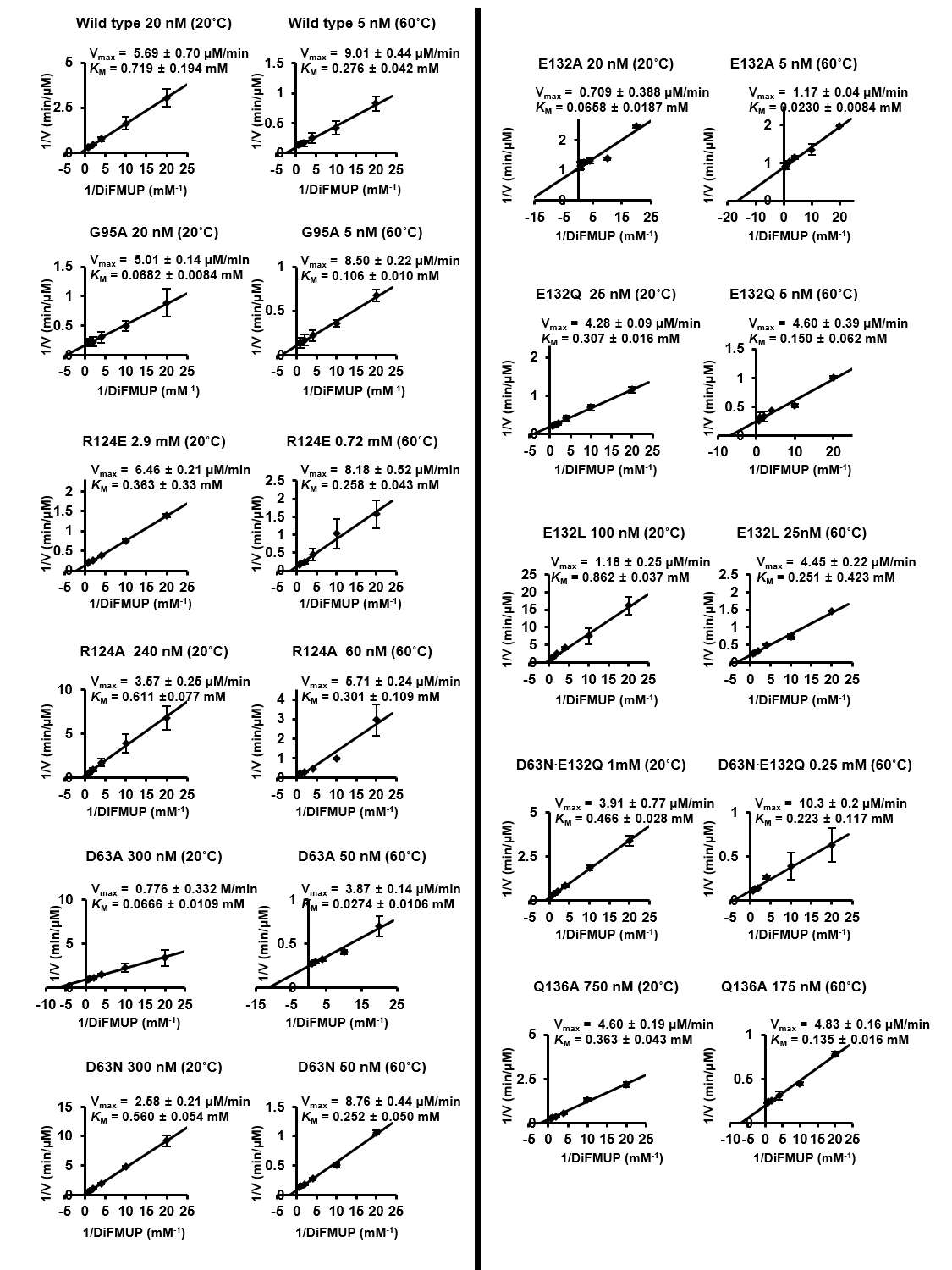

Supplement: S7 Fig — Resulting kinetic parameters including kcat, KM, and kcat/KM values of those proteins listed in Fig 5C. (TIF) [file pone.0197635.s007.tif]

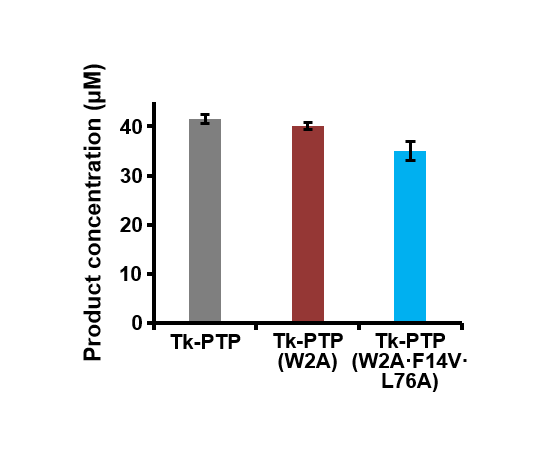

Supplement: S8 Fig — Phosphatase activity assays were carried out at 60°C using wild-type and two mutant Tk-PTP proteins in the same way with those in Fig 5A. (TIF) [file pone.0197635.s008.tif]

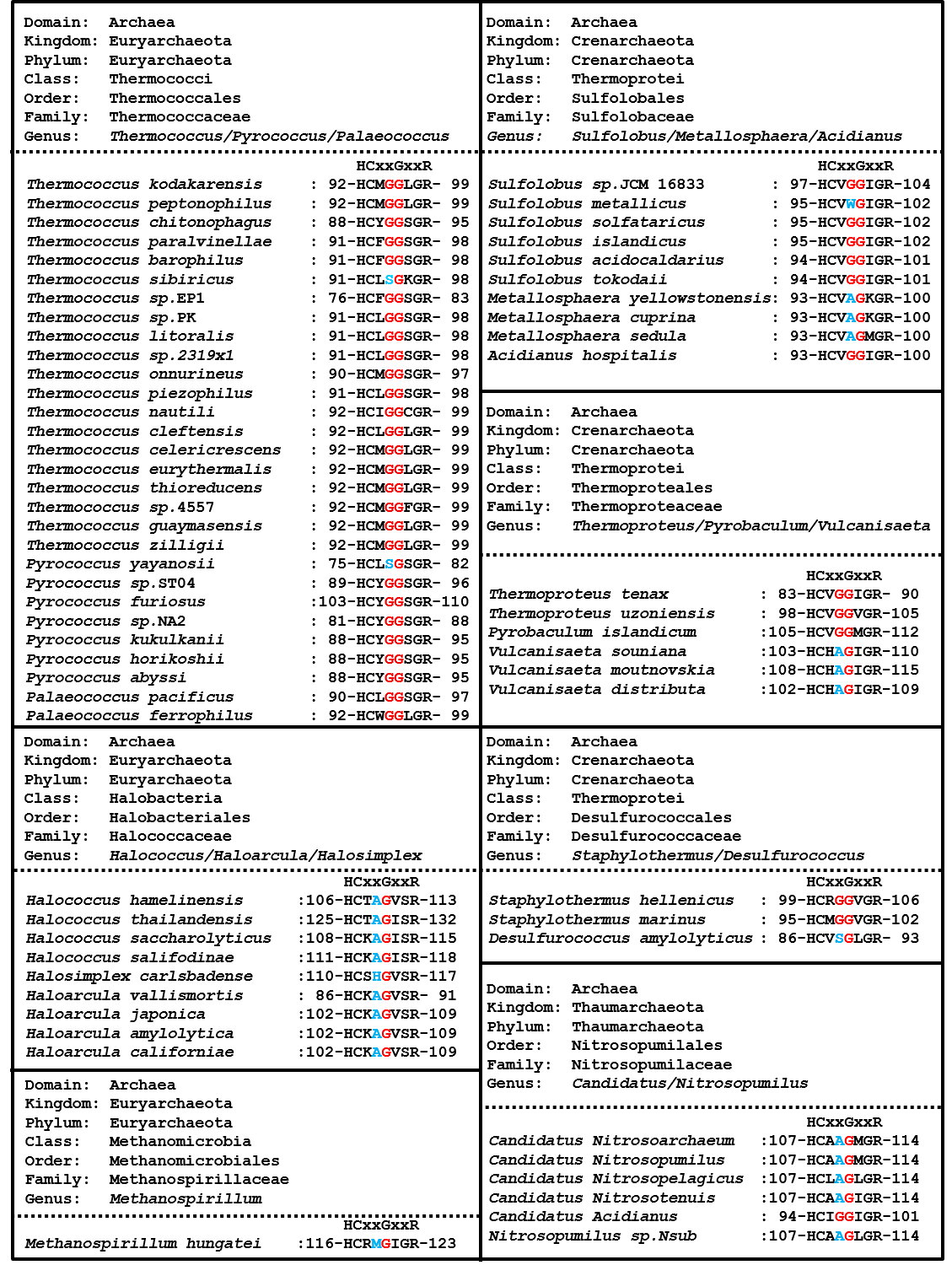

Supplement: S9 Fig — The P-loop residue sequences of 39 putative PTP proteins from euryarchaeota, 19 proteins from crenarchaeota, and 6 proteins from thaumarchaeota kingdoms are aligned. The 4th and 5th residues are marked in red for glycine or in cyan for other residues. (TIF) [file pone.0197635.s009.tif]
